# Supplementary material for: Mind the gap: difference between Framingham heart age and real age increases with age in HIV-positive individuals–a clinical cohort study
Source: BMJ Open. 2013 Oct 25;3(10):e003245. doi: 10.1136/bmjopen-2013-003245 (PMC3808785; doi:10.1136/bmjopen-2013-003245)
Supplement: Author's manuscript [file bmjopen-2013-003245.draft_revisions.pdf]

Mind the gap: difference between Framingham heart age and real age increases with age in HIV-positive individuals: clinical cohort study

Journal:	*BMJ Open*
Manuscript ID:	bmjopen-2013-003245
Article Type:	Research
Date Submitted by the Author:	17-May-2013

Complete List of Authors:	Davies, Teri-Louise; Bristol University, School of Social and Community Medicine<br>Gompels, Mark; North Bristol NHS Trust, HIV Service<br>Johnston, Sarah; North Bristol NHS Trust, HIV Service<br>Bovill, Begoña; North Bristol NHS Trust, HIV Service<br>May, Margaret; University of Bristol, School of Social and Community Medicine

**Primary Subject Heading**:	HIV/AIDS
Secondary Subject Heading:	Cardiovascular medicine
Keywords:	HIV & AIDS < INFECTIOUS DISEASES, Cardiac Epidemiology < CARDIOLOGY, EPIDEMIOLOGY

SCHOLARONE™
Manuscripts

For peer review only

For peer review only - http://bmjopen.bmj.com/site/about/guidelines.xhtml

Mind the gap: difference between Framingham heart age and real age increases with age in HIV-positive individuals: clinical cohort study

Teri-Louise Davies[1], Mark Gompels[2], Sarah Johnston[2], Begoña Bovill[2] and Margaret T. May[1]

[1] School of Social and Community Medicine, University of Bristol (MM Reader, TD PhD student)

[2] HIV Service, North Bristol NHS Trust, Bristol (MG Director and lead clinician, SJ and BB clinicians)

Running title: HIV heart age

Correspondence to:

Dr. Margaret May

Reader in Medical Statistics

School of Social and Community Medicine

University of Bristol

Bristol

BS8 2PS

UK

Telephone: +44 117 9287287

Fax: +44 117 928 7325

m.t.may@bristol.ac.uk

Word Count: 3436, 3 tables, 2 figures, abstract 275 words, 40 references

Key words: HIV, Cardiovascular disease, Framingham risk equation, heart, ageing

For peer review only - http://bmjopen.bmj.com/site/about/guidelines.xhtml

For peer review only

Abstract (275)

Objectives To measure the excess risk of cardiovascular disease (CVD) in HIV-positive individuals by comparing 'heart age' with real age and to estimate associations of patients characteristics with heart age deviation (heart age - real age).

Design Clinical Cohort Study

Setting Bristol HIV clinic, Brecon Unit at Southmead Hospital, Bristol, UK.

Participants 749 HIV-positive adults who attended for care between 2008-2011. Median age was 42 years (IQR 35-49), 67% were male and 82% were treated with antiretroviral therapy.

Main outcome measures We calculated the Framingham 10 year risk of CVD and traced back to 'heart age', the age of an individual with the same score but ideal risk factor values. We estimated the relationship between heart age deviation and real age using fractional polynomial regression. We estimated crude and mutually adjusted associations of sex, age, CD4 count, viral load/treatment status and period of starting antiretroviral therapy with heart age deviation.

Results The average heart age for a male aged 45 years was 48 years for a non-smoker and 60 years for a smoker. Heart age deviation increased with real age and at younger ages was smaller for females than males, although this reversed after age 48 years. Compared to patients with CD4 count <500 cells/mm^3, heart age deviation was 2.4 (95% CI 0.7-4.0) and 4.3 (2.3-6.3) years higher for those with CD4 500-749 cells/mm^3 and ≥750 cells/mm^3 respectively.

Conclusions In HIV-positive individuals, heart age deviation increased with age and CD4 count, which is likely due to higher cholesterol in those with antiretroviral therapy restored CD4 counts. Heart age could be a useful tool to communicate CVD risk to patients and the benefits of stopping smoking.

For peer review only - http://bmjopen.bmj.com/site/about/guidelines.xhtml

Article summary

Article focus

HIV-positive individuals may have a high lifetime risk of cardiovascular disease (CVD) because they are now living to much older ages and have a high prevalence of smoking. The British Heart Foundation has promoted the use of 'heart age', derived from the 10-year Framingham risk equations of general CVD, as a tool for communicating risk to the public and encouraging modification of risk factors. We estimated the difference between real age and 'heart age' in HIV positive individuals and investigated associations of clinical characteristics with higher heart age difference.

Key messages

Our study of people in care for HIV infection in the UK showed that 'heart age' exceeds real age at all ages in men and above age 40 years old in women and is much higher in smokers. Therefore, in HIV-positive individuals, it is important to estimate CVD risk and to intervene on lifestyle factors such as smoking and obesity at young ages because the gap between 'heart age' and real age increases with age.

Strengths and limitations

We were able to estimate Framingham risk and heart age in three-quarters of the patients in the Bristol Cohort. However, some patient groups such as those with a history of injection drug use were under-represented and therefore our results may be more applicable to patients that regularly attend for HIV care. A major limitation of our study is that we did not have information on smoking status. We sought to overcome this by duplicating analyses assuming all were smokers and all were non-

For peer review only - http://bmjopen.bmj.com/site/about/guidelines.xhtml

smokers. We do not yet have full information on CVD events or deaths and so we do not know whether higher estimated CVD risk in this HIV population translates to an elevated rate of CVD.

Introduction

HIV-positive individuals are now living to much older ages [2 3] and therefore may be at high risk of cardiovascular disease (CVD). [4 5] Guidelines for the clinical management of HIV patients stress the importance of assessing risk of CVD and recommend interventions to treat risk factors.[6] Although there have been some attempts to introduce CVD risk scoring tools specifically for HIV-positive individuals,[7-9] none have been independently validated and therefore the Framingham risk equation[10] is still widely used,[11] particularly as the Framingham Heart Study website[12] provides a simple, accessible tool for calculating the risk of developing CVD within 10 years.

Communication of CVD risk to HIV patients is extremely important, particularly the impact of modifiable risk factors, such as smoking. Recently the British Heart Foundation has promoted the use of 'heart age' derived from the 10-year Framingham risk equations for general CVD. [10] A person's 'heart age' is the age of an individual with the same risk score but ideal modifiable risk factor values. Therefore 'heart age' is a useful measure of excess CVD risk adjusted for age and sex.

Our objectives were to compare 'heart age' with actual age and to estimate the association of patient clinical and demographic characteristics with heart age deviation, the difference

For peer review only - http://bmjopen.bmj.com/site/about/guidelines.xhtml

between estimated 'heart age' and their real age, in the Bristol Cohort of HIV-positive individuals.

For peer review only

For peer review only - http://bmjopen.bmj.com/site/about/guidelines.xhtml

Methods

Study participants

The Bristol HIV Cohort study enrols patients attending the Brecon Unit at Southmead Hospital, Bristol, UK. Routine clinical data collected on patients attending for HIV care up to November 2011were available for analysis as part of the UK CHIC study. In accordance with data protection policy, all data were anonymised. Included patients were aged 18 years and over and were not infected peri-natally.

Data measurement and availability

Demographic data on sex, date of birth, ethnicity (black African, white and other), assumed HIV transmission group and the dates of HIV diagnosis and first clinic visit were available. CD4 cell count and HIV-1 RNA were usually measured at each clinic visit. Details of antiretroviral therapy (ART) and non-HIV medications were available. Systolic blood pressure (SBP) and total and HDL cholesterol have been measured since 2008, at first visit and at least annually thereafter according to protocol. Patients ever recorded as taking anti-hypertensive medication were classed as treated for high blood pressure. Diagnosis of diabetes mellitus was recorded in patient notes. Patients with missing diabetes status were assumed to be non-diabetic. Smoking status was not available. Patients included in analyses had at least one set of Framingham risk factors (SBP, total and HDL cholesterol), CD4 count and HIV-1 RNA measured within a 6 month time window. We used the latest available measurements to calculate the Framingham risk score.

Statistical methods

Calculation of Framingham 10-year risk of CVD and heart age

For peer review only - http://bmjopen.bmj.com/site/about/guidelines.xhtml

For peer review only

We calculated the 10-year risk of CVD for each person using the sex-specific Framingham equations for general CVD[10 12] which include age, total and HDL cholesterol, SBP, treatment for hypertension, smoking and diabetes status. We used the Framingham risk to trace back to 'heart age': the age of an individual with the same score but ideal risk factor values (non-smoker, non-diabetic, untreated SBP 125 mmHg, total cholesterol 180 mg/dL, HDL 45 mg/dL[10]). For example, if a 40 year old male has a 10-year CVD risk of 5.6%, his 'heart age' would be 45 years because a 45 year old male with ideal risk factors has a 10-year risk of CVD of 5.6%. For comparison, the 10-year CVD risk for a 40 year old male with ideal risk factors is 3.9%. Data on smoking status was not collected in the Bristol cohort and therefore analyses were conducted twice, firstly assuming all were smokers, and secondly assuming all non-smokers. Heart age deviation ('heart age' - real age) was calculated for each individual for each smoking assumption.

<u>Analysis of heart age deviation</u>

We estimated the difference between age and 'heart age' overall and by age group (18-39, 40-49, 50-59 and ≥ 60). We used box plots to compare the distribution of 'heart age' with median real age for male and female smokers and non-smokers stratified by real age group. We used fractional polynomial regression models[13] separately for male and female smokers and non-smokers to show the variation of heart age deviation with age. We used univariable and multivariable linear regression models to estimate crude and mutually adjusted associations of sex, age group, current CD4 count, treatment/viral load status (untreated, treated and suppressed, treated and not suppressed) and period of starting ART (pre v. post 2003) with heart age deviation. We also considered models that included duration since HIV diagnosis, duration since first clinic visit, duration and type of ART.

For peer review only - http://bmjopen.bmj.com/site/about/guidelines.xhtml

In sensitivity analyses, we repeated the main analyses firstly including only those on ART and secondly restricting to males. We also tested whether CD4 count was associated with total cholesterol, the ratio of total to HDL cholesterol and SBP, controlling for age and sex. Results are presented as the difference between heart age and real age in years with 95% confidence intervals. All calculations were executed in STATA version 12.1 [14].

Results

Of the 1,013 patients who attended the clinic, 749 (74%) had measurements of CD4 count, HIV-1 RNA, total and HDL cholesterol, SBP taken within a 6 month period. Patient demographic and clinical characteristics at the time of the Framingham risk assessment are shown in table 1. Two thirds of the patients were male, the majority of whom were men who have sex with men (MSM) and of white ethnicity. In contrast, the majority of females (63%) were of Black African origin. Compared to those included in analyses, excluded individuals (N=264) without risk factor measurements were similar in age, sex and ethnicity, but were twice as likely to be infected via injection drug use (IDU), blood product or “unknown” risk group. The median latest CD4 count was 484 (IQR 322-657) mm^3 in those excluded which was comparable with the treated and untreated included patients (table 1).

Table 2 shows real age compared with heart age and estimated heart age deviation overall and by age group, stratified by sex and smoking status. Heart age was greater than real age for all groups except for non-smoking females aged 18-39 years. The mean age of the men was 44.3 years and their mean heart age was 47.7 years if a non-smoker or 59.2 years if a smoker. The females were slightly younger with a mean age of 40.6 years and corresponding mean heart ages of 42.4 and 53.1 years assuming non-smoker and smoker respectively.

For peer review only - http://bmjopen.bmj.com/site/about/guidelines.xhtml

Figure 1 illustrates the distribution of heart age by age group. The box represents the middle 50% of the distribution with the median marked as a line, and the tails extend to 95% of the distribution with outliers marked as dots. There was an increasing trend across age groups in the deviation between the median age and median heart age.

Figure 2 shows heart age deviation increased substantially with real age and was much higher for smokers eg for males aged 50, the heart age deviation was around 18 years in smokers and around 5 years in non-smokers (illustrated by green line). At younger ages, females had smaller heart age deviation than males, but this reversed after about age 48. However, the deviation is relative to sex-specific risk in those with ideal risk factors and females have lower absolute risk than males in the general population.

The crude and adjusted associations of variables with heart age deviation are shown in table 3. Duration since HIV diagnosis, duration since first clinic visit, and duration of ART were not associated with heart age deviation. Compared with ages 18-39, those aged ≥ 60 years had an increase of 8.87 (95% CI 5.90 to 11.84) years in heart age deviation, and this was approximately doubled in smokers. Compared with those with CD4 count <500 cells/mm^3, those with CD4 count ≥ 750 cells/mm^3 had an increase of 4.28 (95% CI 2.28 to 6.27) years in heart age deviation and this effect was independent of smoking status. When analyses were restricted to males only, the associations of age and CD4 count with heart age deviation were somewhat weaker. Patterns of results were similar when analyses were restricted to individuals on ART. There was no evidence of a difference in heart age deviation between those on PI- compared with NNRTI-based ART at the time of the CVD risk assessment. Higher total cholesterol (adjusted for age and sex), was associated with higher CD4 count (80 mg/dL increase in cholesterol per 50 increase in CD4 count, p=0.01) and this was not

For peer review only - http://bmjopen.bmj.com/site/about/guidelines.xhtml

attenuated by adjusting for HDL cholesterol. Higher total:HDL cholesterol was similarly associated with higher CD4 count. SBP was not associated with CD4 count. There was some evidence that treated patients with unsuppressed virus, but not untreated patients, had greater heart deviation than those with suppressed virus.

Discussion

Main results

We showed that in the Bristol HIV cohort on average 'heart age' was greater than real age for men of all ages and for women aged over 40 years old. Heart age deviation widened with increasing age and was very much higher if people smoke. On average, a 45 year-old male smoker had a 'heart age' of around 60 years. Our results suggest that in women the difference between age and 'heart age' increased steeply after menopausal age. Untreated patients and treated patients who were virally suppressed had similar heart ages, but those who were not virally suppressed on ART had higher heart age deviation. Higher CD4 count was associated with higher heart age deviation.

Strengths and limitations

As far as we are aware, this is the first study to calculate 'heart age' based on the Framingham CVD risk score for HIV-positive individuals. Complete data on Framingham risk factors and HIV biomarkers were only available on 74% of the patients in the Bristol Cohort and some patient groups such as IDU were under-represented. Therefore our results may be more applicable to patients that regularly attend for HIV care. Measurement error and misclassification may have biased our results. We only used one cross-sectional assessment of Framingham CVD risk score and cholesterol measurements were not all fasting measures.

For peer review only - http://bmjopen.bmj.com/site/about/guidelines.xhtml

For peer review only

We assumed that individuals without diabetes status recorded were not diabetic and that those who had ever been prescribed medication for high blood pressure remained treated which may have resulted in some misclassification. A major limitation of our study is that we did not have information on smoking status. We sought to overcome this by duplicating analyses assuming all were smokers and all were non-smokers. Our results may be biased if smoking is associated with changes in other risk factors such as SBP and cholesterol that are intermediate in the pathway from smoking to CVD. Our study was limited to cross-sectional analysis and therefore does not show within person changes in CVD risk. We do not yet have full information on CVD events or deaths and so we do not know whether higher estimated CVD risk in this HIV population translates to an elevated rate of CVD. A much larger number of patients would be required to properly analyse CVD events and causes of death. The calculation of "heart age" uses a set of "ideal" risk factor values proposed by the Framingham investigators. It is likely that "normal" or "average" CVD risk factor values in the UK general population are somewhat worse than the "ideal". According to the Health Survey for England 2006 which reported CVD risk factors in adults, for males aged >35 years the mean total and HDL cholesterol were 220mg/dl and 50mg/dl respectively, somewhat higher than the ideal values of 180mg/dl and 45mg/dl, and only 69% had SBP<140 and DBP<90 mmHg without medication.[1] Unfortunately, we did not have an age and sex matched HIV negative population for direct comparison.

In context with other studies

Our finding that heart age was greater than real age for the majority of HIV-positive individuals in this study population is concordant with a study that found increased CVD rates among HIV-positive compared with negative controls.[15] The increased burden of CVD

For peer review only - http://bmjopen.bmj.com/site/about/guidelines.xhtml

among HIV-positive individuals is likely a consequence of increased traditional risk factors, including dyslipidemia and insulin resistance, and non-traditional risk factors such as immune activation and inflammation that may contribute to the accelerated ageing process characterised by higher than expected rates of non-infectious co-morbidities.[16] Higher prevalence of smoking also contributes to the CVD epidemic in the HIV-positive population[17 18] as may the use of recreational drugs. ART itself may contribute to CVD, but no effect of current PI use was detected in this UK cohort.

We found that heart age deviation was greater at older ages. This is particularly significant as the mean age of HIV infection is increasing and it is predicted that by 2020 in the USA 50% of people living with HIV will be over 50 years old.[19] Although our study was cross-sectional, it is likely that the gap between real and heart age increases within individuals as they age. Older HIV-positive individuals, compared with matched controls, have been found to have a higher prevalence of hypertension, hypertriglyceridemia , low bone density and lipodystrophy suggesting that HIV and treatment related factors accelerate normal ageing.[4]

Our finding that higher current CD4 count was associated with higher estimated Framingham risk of CVD is to be interpreted with caution since the SMART study of structured treatment interruption based on CD4 count found that CVD events were greater in those with lower CD4.[20] However, our results are in line with another study that found a higher prevalence of clinically evident lipodystrophy and higher CD4 cell counts in patients with higher Framingham score.[21] A study that used cardiac computed tomography imaging to identify coronary artery calcium found that vascular age was increased in over 40% of patients, with an average increase of 15 years over the chronological age, also found that current CD4 count was associated with higher vascular age.[22] Atherosclerosis is an inflammatory process of the

For peer review only - http://bmjopen.bmj.com/site/about/guidelines.xhtml

subintimal layer of the arterial wall in which lymphocytes and macrophages play a major role. The CD4+ type 1 T helper (Th1) lymphocyte is the predominant subtype of T cells in atherosclerotic plaques of humans.[23 24] Furthermore it has been demonstrated in a mouse model that CD4 cells play a pathogenic role in atherosclerosis.[25] Therefore ART-induced increase in CD4 count may contribute to the development of atherosclerosis in HIV-positive patients. Other studies have shown that low CD4 count (<350cell/ml) is associated with higher rates of CVD or subclinical atherosclerosis. It may be that the association of CD4 with CVD is a U-shaped curve with low CD4 associated with acute inflammatory processes and high CD4 associated with chronic ongoing inflammatory processes.[22] However, in our study the association of CD4 with heart age may be mediated through components of the Framingham risk score, such as SBP or cholesterol.

Risk prediction

Although some HIV-specific coronary heart disease (CHD) and CVD prediction algorithms have been proposed,[7 9] none have been externally validated by independent data and the Framingham risk score is still widely used.[11 26 27] Risk prediction in HIV-positive populations firstly focused on CHD,[8] but now the importance of risk assessment for CVD has been recognised by guidelines.[6] D'Agostino summarised the state of CVD risk prediction as applied to HIV populations in a review article.[27] Studies in HIV populations have compared the degree of correlation of 3 traditional risk prediction algorithms, Framingham, Systematic Coronary Risk Evaluation (SCORE) and Prospective Cardiovascular Munster (PROCAM) equations.[28-30]. The estimation of relative effects of traditional risk factors on CVD outcomes appears similar between HIV-positive and HIV negative individuals.[31] However, it may be that HIV-specific risk equations that include HIV-specific risk factors may perform better

For peer review only - http://bmjopen.bmj.com/site/about/guidelines.xhtml

than existing algorithms because of potential differences in etiology of CVD in the HIV population. For example, D-dimer, a marker of inflammation, has been found to be independently predictive of CVD events. [32] However, the Framingham risk score may partially capture inflammation since markers of inflammation have been found to be associated with a higher score in HIV patients compared with controls [33] and the score has been shown to correlate with the presence of subclinical atherosclerosis measured by carotid artery intima-media thickness in HIV-positive individuals.[34 35] Atherosclerosis may also be high in untreated patients, supporting a role of HIV infection itself as a risk factor.[36]

The ability to accurately predict CVD risk is an essential element of this population's care. The Framingham equation for CHD predicted well in The Data Collection on Adverse events of Anti-HIV Drugs Study (D:A:D) in terms of discrimination, but tended to underestimate risk in smokers.[8] The Framingham risk scores may require recalibration to adjust for over or under prediction in the HIV population.[37 38] Factors unique to HIV, such as effects of different antiretroviral drugs, may influence the performance of standard risk prediction tools, as they may change CVD risk both through alterations of traditional risk factors and by contributing to inflammatory and immunologic risk factors. D:A:D found both observed and predicted rates of MI increased with time on ART implying that ART-induced changes in conventional risk factors at least partially explained the increase in risk of MI.[8] The D:A:D predictive model for CVD tailored to HIV patients included exposure to indinavir, boosted lopinavir and abacavir as well as the traditional Framingham risk factors.[9]

The HIV-HEART study found that not only were CVD risk factors high in the HIV population, but also that they are under-treated and therefore risk factor management of HIV

For peer review only - http://bmjopen.bmj.com/site/about/guidelines.xhtml

patients requires further improvement.[26] The European AIDS Clinical Society (EACS) guidelines on the prevention and management of metabolic disease in HIV [6] state that CVD risk should be assessed in HIV-positive individuals at regular intervals. They recommend lifestyle interventions should focus on counselling to stop smoking, modify diet and take regular exercise. A healthy diet, exercise and maintaining normal body weight tend to reduce dyslipidemia.

Smoking

Increased Framingham risk scores have been found in HIV-positive compared with negative controls, but this has mostly been due to higher prevalence of smoking rather than to higher cholesterol.[39] Smoking is the most important modifiable risk factor. A pilot study of a smoking cessation programme using counselling and nicotine replacement therapy in the Swiss HIV Cohort Study [40] found that implementing a smoking cessation programme was feasible in HIV-positive individuals. The D:A:D study found that the adjusted incidence rate ratio of CVD decreased from 2.32 within the first year of stopping smoking to 1.49 after greater than 3 years compared with those who never smoked. A recent study from Denmark estimated that HIV-positive individuals lose more years from smoking than from HIV infection.[18]

Future work

In order for accurate CVD risk assessment to be carried out, HIV cohorts need to collect better information on Framingham risk factors and, in particular, on current smoking status of patients. Our study should be considered as a pilot study for assessing heart age. Ideally, this

For peer review only - http://bmjopen.bmj.com/site/about/guidelines.xhtml

type of analysis would need to be rolled out into a much larger national cohort to properly gauge which factors predict heart age deviation. In addition, CVD events and death would allow us to test whether the Framingham equations are transferable to the UK HIV population. We need to assess the use of heart age as a communication tool for behaviour intervention. More research is required to investigate whether Framingham risk estimates accurately translate to actual CVD events and deaths or whether including HIV specific risk factors in the CVD risk equations might result in a more accurate prediction tool for this population.

Implications and conclusion

HIV-positive individuals in this cohort had a considerably increased risk of CVD compared with the ideal reference values. Our research, which showed that heart age exceeds real age at all ages in men and above age 40 years old in women, implies that it is important to estimate CVD risk in HIV-positive individuals. Furthermore, since the gap between heart age and real age increased as people get older, it is important to intervene on lifestyle factors such as smoking and obesity at young ages. Cardiovascular risk expressed in terms of an individual's 'heart age deviation', rather than absolute risk of CVD, may have more impact on patients in programmes aiming to intervene on risk factors. Heart age may be a particularly useful tool in communicating risk to younger patients who are at low absolute risk of CVD. Tracking change in heart age, such as that due to smoking cessation, may provide strong motivation towards life style changes.

For peer review only - http://bmjopen.bmj.com/site/about/guidelines.xhtml

Acknowledgements: We thank all patients, Susan Allan, and the team at Southmead Hospital, Bristol, UK. This work was presented in part at the Eleventh International Congress on Drug Therapy in HIV Infection Glasgow, UK 11-15 November 2012. Abstract available: Davies T et al. Journal of the International AIDS Society 2012, 15(Suppl 4):18152

http://www.jiasociety.org/index.php/jias/article/view/18152 | http://dx.doi.org/10.7448/IAS.15.6.18152

Contributors: MG and MM contributed to study design and interpretation of analyses. TD analysed the data, compiled the tables and graphs and wrote the first draft of the paper. MM supervised statistical analyses. All authors contributed to the writing of the paper, edited, revised and approved the final version of the paper and had full access to all the data in the study. MM had the final responsibility for the decision to submit for publication and is the guarantor.

Funding: Margaret May is funded by the Medical Research Council, UK (grant MR/J002380/1). Teri Davies is funded by the UK Medical Research Council as a PhD student in the Bristol Centre for Systems Biomedicine (BCSBmed) doctoral training centre. The views expressed in this manuscript are those of the researchers and not necessarily those of the Medical Research Council. The funders had no role in carrying out the study or in the decision to submit the manuscript for publication.

Competing interests: none.

Ethical approval: The project was approved by a Multi-centre Research Ethics Committee and by local ethics committees.

Copyright: The Corresponding Author has the right to grant on behalf of all authors and does grant on behalf of all authors, an exclusive licence (or non exclusive for government employees) on a worldwide basis to the BMJ Publishing Group Ltd to permit this article (if accepted) to be published in BMJ editions and any other BMJPGL products and sublicences such use and exploit all subsidiary rights, as set out in our licence.

For peer review only - http://bmjopen.bmj.com/site/about/guidelines.xhtml

Data sharing: no additional data available.

For peer review only - http://bmjopen.bmj.com/site/about/guidelines.xhtml

Table 1: Patient demographic and clinical characteristics at time of Framingham Score assessment

	Number of Patients N = 749	Percent
**Sex**		
Male	503	67.2
**Transmission risk group**		
Heterosexual	386	51.5
MSM	312	41.7
IDU	23	3.1
Blood Product	7	0.9
Unknown	21	2.8
**Ethnicity**		
Black African	228	30.4
White	441	58.9
Other	80	10.7
**Age (years) (median, IQR)**	42.2	(35.5-49.4)
**Age category (years)**		
18-39	310	41.4
40-49	265	35.4
50-59	118	15.8
60+	56	7.5
**Treatment status**		
On ART	612	81.7
**Started ART pre 01/01/2003**	168	22.4
**Viral load (copies per ml)**		
Treated and suppressed (vl ≤ 50)	535	71.4
Treated and unsuppressed	77	10.3
Untreated	137	18.3
**Median cd4 count (mm^3), untreated (IQR)**	477	(334-602)
**Median cd4 count (mm^3), treated (IQR)**	559.5	(409-721)
**cd4 count (mm^3)**		
<500	318	42.5
500 - 749	275	36.7
≥ 750	156	20.8
**Median total cholesterol(mg/dL), (IQR)**	189.1	(162-220)
**Median HDL cholesterol(mg/dL), (IQR)**	50.2	(38.6-61.8)
**Median systolic blood pressure(mmHg), (IQR)**	131	(117-143)

For peer review only - http://bmjopen.bmj.com/site/about/guidelines.xhtml

For peer review only

HIV heart age

Table 2: Age, heart age and heart age deviation (heart age- real age) by age group for male and female smokers and non-smokers.

sex	smoking assumption	real age category (years)	n (%)	mean real age (years)	mean heart age (years)	deviation=heart age-real age (years) mean	(95% CI)
males	non-smoker	18-39	184(37%)	33.5	34.9	1.3	(0.60,2.09)
		40-49	182 (36%)	45.1	48.5	3.3	(2.17,4.53)
		50-59	91 (18%)	53.4	59.5	6.1	(3.85,8.37)
		60-	46 (9%)	66.2	73.0	6.8	(2.19,11.36)
		total	503(100%)	44.3	47.7	3.4	(2.65,4.21)
males	smoker	18-39	184(37%)	33.5	43.3	9.8	(8.78,10.74)
		40-49	182 (36%)	45.1	60.1	15.0	(13.53,16.48)
		50-59	91 (18%)	53.4	73.9	20.4	(17.64,23.24)
		60-	46 (9%)	66.2	89.6	23.3	(18.35,28.29)
		total	503(100%)	44.3	59.2	14.8	(13.82,15.84)
females	non-smoker	18-39	126(51%)	33.1	31.7	-1.4	(-2.93,0.05)
		40-49	83 (34%)	44.1	47.1	3.0	(-0.13,6.10)
		50-59	27 (11%)	55.2	62.9	7.7	(1.54,13.93)
		60-	10 (4%)	65.9	82.2	16.3	(0.07,32.52)
		total	246(100%)	40.6	42.4	1.8	(0.15,3.41)
females	smoker	18-39	126(51%)	33.1	39.9	6.8	(4.87,8.64)
		40-49	83 (34%)	44.1	59.2	15.0	(11.19,18.90)
		50-59	27 (11%)	55.2	78.9	23.7	(15.77,31.55)
		60-	10 (4%)	65.9	99.8	33.9	(16.79,51.01)
		total	246(100%)	40.6	53.1	12.5	(10.42,14.61)

For peer review only

For peer review only - http://bmjopen.bmj.com/site/about/guidelines.xhtml

HIV heart age

Table 3: Crude and mutually adjusted heart age deviation (difference from comparator group*) according to patient characteristics for non-smokers and smokers

	All individuals (N=749)			
	Non smoker Deviation (years) (95% CI)		Smoker Deviation (years) (95% CI)	
**Variable**	**Crude**	**Adjusted**	**Crude**	**Adjusted**
**Female vs Male**	-1.65 (-3.25,-0.05)	-0.96 (-2.52,0.59)	-2.32 (-4.38,-0.25)	-0.72(-2.62,1.18)
**Age category**	-	-	-	-
18-39*	-	-	-	-
40-49	3.02 (1.35,4.69)	3.38 (1.66,5.10)	6.48(4.44,8.52)	6.75(4.65,8.86)
50-59	6.27(4.11,8.43)	6.39(4.17,8.61)	12.64(10.00,15.27)	12.64(9.94,15.35)
60+	8.26(5.36,11.16)	8.87(5.90,11.84)	16.67(13.13,20.20)	17.21(13.59,20.83)
**CD4 count category (cells/mm^3)**	-	-	-	-
<500*	-	-	-	-
500-750	1.91(0.23,3.60)	2.39(0.74,4.03)	2.50(0.34,4.66)	3.01(1.00,5.02)
>=750	4.23(2.23,6.23)	4.28(2.28,6.27)	6.12(3.55,8.68)	5.44(3.01,7.87)
**Viral load category**	-	-	-	-
Treated with suppressed vl*	-	-	-	-
Treated with unsuppressed vl	1.07(-1.44,3.59)	2.84(0.40,5.29)	-0.19(-3.42,3.04)	2.90(-0.08,5.89)
Untreated	-1.39(-3.36,0.58)	0.27(-1.74,2.27)	-3.61(-6.15,-1.07)	-0.28(-2.73,2.17)
**ART start date pre 01/01/2003**	0.18(-1.62,1.99)	-2.28(-4.14,-0.42)	1.98(-0.35,4.30)	-2.43(-4.70,-0.17)
**Constant**	-	-1.26(-3.00,0.49)	-	6.70(4.57,8.82)

For peer review only - http://bmjopen.bmj.com/site/about/guidelines.xhtml

HIV heart age

Figure 1: Box plots of distribution of heart age by age group for female non-smokers (upper left), female smokers (upper right), male non-smokers (lower left), male smokers (lower right). The median real age for each age group is shown in red for comparison. In the box plot, the whiskers include 95% of the distribution and the box includes the middle 50% with the median marked as a line. Outliers are shown as filled circles.

For peer review only

For peer review only - http://bmjopen.bmj.com/site/about/guidelines.xhtml

HIV heart age

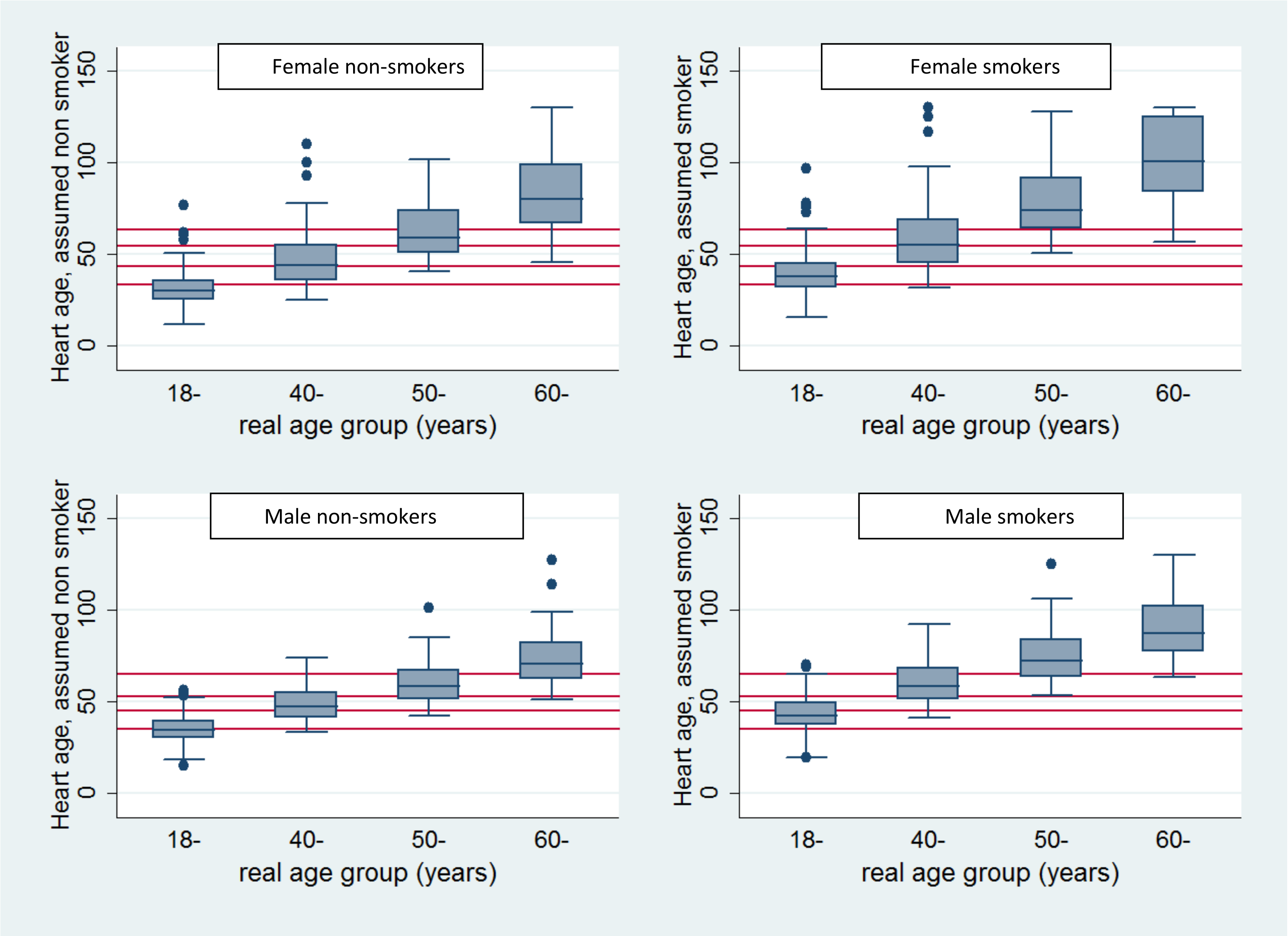

For peer review only - http://bmjopen.bmj.com/site/about/guidelines.xhtml

Figure 2: Heart age deviation in years (95% CI shown shaded in gray) for male and female smokers and non-smokers for real ages 20 to 70 years old

For peer review only

For peer review only - http://bmjopen.bmj.com/site/about/guidelines.xhtml

HIV heart age

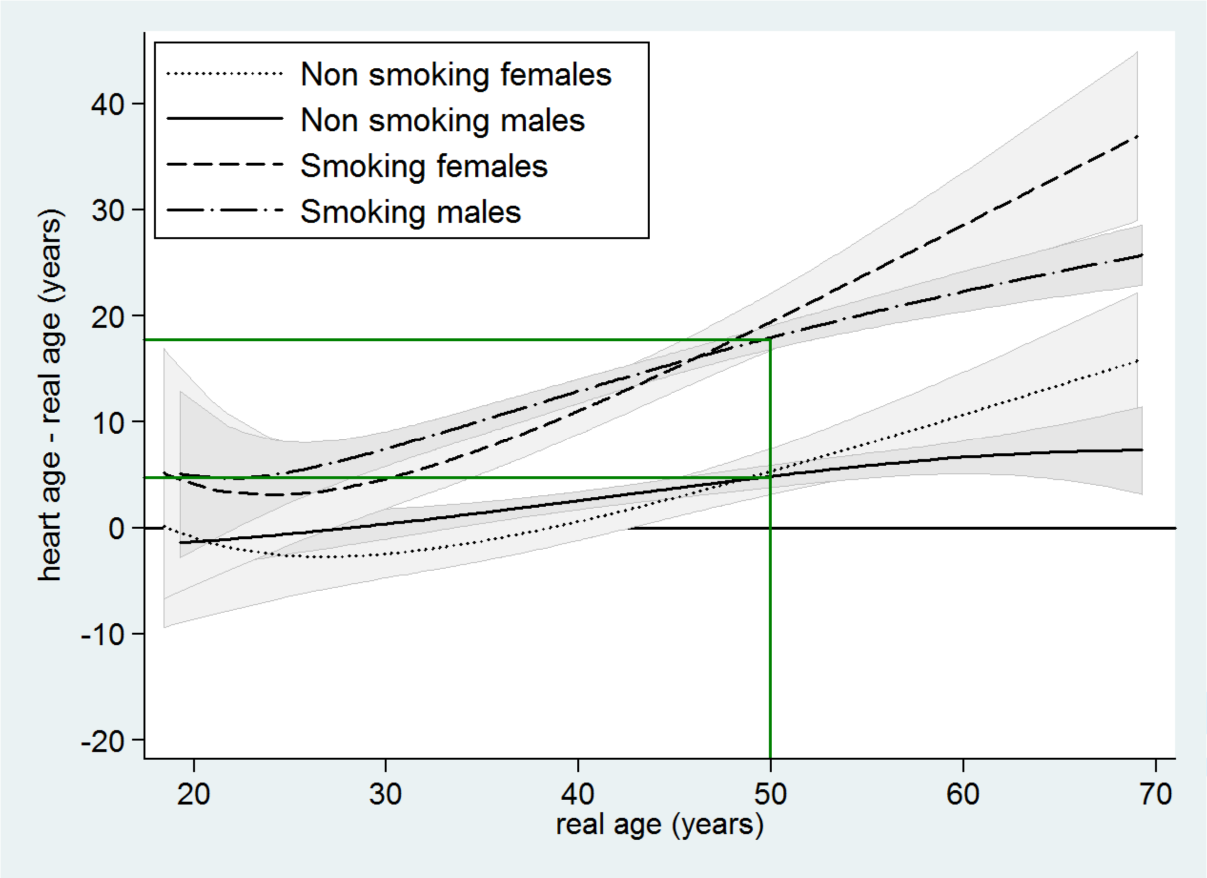

For peer review only - http://bmjopen.bmj.com/site/about/guidelines.xhtml

REFERENCES

1. of NCfSRNatD, School EaPHatRFaUCM, (UCL). Health Survey for England - 2006, CVD and risk factors for adults, obesity and risk factors for children. In: R. C, J. M, editors, 2008.
2. May MT, Ingle SM. Life expectancy of HIV-positive adults: a review. *Sex Health* 2011;8(4):526-33.
3. Nakagawa F, Lodwick RK, Smith CJ, Smith R, Cambiano V, Lundgren J, et al. Projected life expectancy of people with HIV according to timing of diagnosis. *AIDS* 2012;26:335-43.
4. Onen NF, Overton ET, Seyfried W, Stumm ER, Snell M, Mondy K, et al. Aging and HIV infection: a comparison between older HIV-infected persons and the general population. *HIV clinical trials* 2010;11(2):100-9.
5. Law M, Friis-Moller N, Weber R, Reiss P, Thiebaut R, Kirk O, et al. Modelling the 3-year risk of myocardial infarction among participants in the Data Collection on Adverse Events of Anti-HIV Drugs (DAD) study. *HIV.Med* 2003;4(1):1-10.
6. Lundgren JD, Battegay M, Behrens G, De Wit S, Guaraldi G, Katlama C, et al. European AIDS Clinical Society (EACS) guidelines on the prevention and management of metabolic diseases in HIV. *HIV Med* 2008;9(2):72-81.
7. May M, Sterne JA, Shipley M, Brunner E, d'Agostino R, Whincup P, et al. A coronary heart disease risk model for predicting the effect of potent antiretroviral therapy in HIV-1 infected men. *International journal of epidemiology* 2007;36(6):1309-18.
8. Law MG, Friis-Moller N, El-Sadr WM, Weber R, Reiss P, D'Arminio MA, et al. The use of the Framingham equation to predict myocardial infarctions in HIV-infected patients: comparison with observed events in the D:A:D Study. *HIV.Med.* 2006;7(4):218-30.
9. Friis-Moller N, Thiebaut R, Reiss P, Weber R, Monforte AD, De WS, et al. Predicting the risk of cardiovascular disease in HIV-infected patients: the Data collection on Adverse Effects of Anti-HIV Drugs Study. *Eur J Cardiovasc Prev Rehabil.* 2010.
10. D'Agostino RB, Sr., Vasan RS, Pencina MJ, Wolf PA, Cobain M, Massaro JM, et al. General cardiovascular risk profile for use in primary care: the Framingham Heart Study. *Circulation* 2008;117(6):743-53.
11. Burke EG, Nelson J, Kwong J, Cook PF. Cardiovascular risk assessment for persons living with HIV. *The Journal of the Association of Nurses in AIDS Care : JANAC* 2012;23(2):134-45.
12. Study; FH. General Cardiovascular Disease (10-year risk), 2011.
13. Royston P, Ambler G, Sauerbrei W. The use of fractional polynomials to model continuous risk variables in epidemiology. *Int.J.Epidemiol.* 1999;28(5):964-74.
14. Stata Statistical Software: Release 12 [program]. College Station, TX: StataCorp LP., 2011.
15. Triant VA, Lee H, Hadigan C, Grinspoon SK. Increased acute myocardial infarction rates and cardiovascular risk factors among patients with human immunodeficiency virus disease. *The Journal of clinical endocrinology and metabolism* 2007;92(7):2506-12.
16. Petoumenos K, Worm SW. HIV infection, aging and cardiovascular disease: epidemiology and prevention. *Sex Health* 2011;8(4):465-73.
17. Smith CJ, Levy I, Sabin CA, Kaya E, Johnson MA, Lipman MC. Cardiovascular disease risk factors and antiretroviral therapy in an HIV-positive UK population. *HIV Med* 2004;5(2):88-92.
18. Helleberg M, Afzal S, Kronborg G, Larsen CS, Pedersen G, Pedersen C, et al. Mortality Attributable to Smoking Among HIV-1-Infected Individuals: A Nationwide, Population-Based Cohort Study. *Clin Infect Dis* 2013;56(5):727-34.
19. Neergaard L. Aging AIDS epidemic raises new questions. *NBC News*, 2012.
20. El-Sadr WM, Lundgren JD, Neaton JD, Gordin F, Abrams D, Arduino RC, et al. CD4+ count-guided interruption of antiretroviral treatment. *N.Engl.J.Med.* 2006;355(22):2283-96.
21. De Socio GV, Martinelli C, Ricci E, Orofino G, Valsecchi L, Vitiello P, et al. Relations between cardiovascular risk estimates and subclinical atherosclerosis in naive HIV patients: results from the HERMES study. *Int J STD AIDS* 2010;21(4):267-72.
22. Guaraldi G, Zona S, Alexopoulos N, Orlando G, Carli F, Ligabue G, et al. Coronary aging in HIV-infected patients. *Clin Infect Dis* 2009;49(11):1756-62.

For peer review only - http://bmjopen.bmj.com/site/about/guidelines.xhtml

23. Frostegard J, Ulfgren AK, Nyberg P, Hedin U, Swedenborg J, Andersson U, et al. Cytokine expression in advanced human atherosclerotic plaques: dominance of pro-inflammatory (Th1) and macrophage-stimulating cytokines. *Atherosclerosis* 1999;145(1):33-43.
24. Maggi P, Perilli F, Lillo A, Carito V, Epifani G, Bellacosa C, et al. An ultrasound-based comparative study on carotid plaques in HIV-positive patients vs. atherosclerotic and arteritis patients: atherosclerotic or inflammatory lesions? *Coronary artery disease* 2007;18(1):23-9.
25. Zhou X, Nicoletti A, Elhage R, Hansson GK. Transfer of CD4(+) T cells aggravates atherosclerosis in immunodeficient apolipoprotein E knockout mice. *Circulation* 2000;102(24):2919-22.
26. Reinsch N, Neuhaus K, Esser S, Potthoff A, Hower M, Mostardt S, et al. Are HIV patients undertreated? Cardiovascular risk factors in HIV: results of the HIV-HEART study. *European journal of preventive cardiology* 2012;19(2):267-74.
27. D'Agostino RB, Sr. Cardiovascular risk estimation in 2012: lessons learned and applicability to the HIV population. *The Journal of infectious diseases* 2012;205 Suppl 3:S362-7.
28. Knobel H, Jerico C, Montero M, Sorli ML, Velat M, Guelar A, et al. Global cardiovascular risk in patients with HIV infection: concordance and differences in estimates according to three risk equations (Framingham, SCORE, and PROCAM). *AIDS patient care and STDs* 2007;21(7):452-7.
29. Guimaraes MMM, Greco DB, Garces AHI, de Oliveira AR, Foscolo RB, Machado LJD. Coronary heart disease risk assessment in HIV-infected patients: a comparison of Framingham, PROCAM and SCORE risk assessment functions. *Int J Clin Pract* 2010;64(6):739-45.
30. Barros ZM, de Alencar Ximenes RA, Miranda-Filho DB, de Albuquerque Mde F, Melo HR, Carvalho EH, et al. Comparison between the Framingham and prospective cardiovascular of Munster scores for risk assessment of coronary heart disease in human immunodeficiency virus-positive patients in Pernambuco, Brazil. *Metabolic syndrome and related disorders* 2010;8(6):489-97.
31. Schambelan M, Wilson PW, Yarasheski KE, Cade WT, Davila-Roman VG, D'Agostino RB, Sr., et al. Development of appropriate coronary heart disease risk prediction models in HIV-infected patients. *Circulation* 2008;118(2):e48-53.
32. Ford ES, Greenwald JH, Richterman AG, Rupert A, Dutcher L, Badralmaa Y, et al. Traditional risk factors and D-dimer predict incident cardiovascular disease events in chronic HIV infection. *AIDS* 2010;24(10):1509-17.
33. Guzman-Fulgencio M, Medrano J, Rallon N, Echeverria-Urabayen A, Benito JM, Restrepo C, et al. Soluble markers of inflammation are associated with Framingham scores in HIV-infected patients on suppressive antiretroviral therapy. *J Infection* 2011;63(5):382-90.
34. Parra S, Coll B, Aragones G, Marsillach J, Beltran R, Rull A, et al. Nonconcordance between subclinical atherosclerosis and the calculated Framingham risk score in HIV-infected patients: relationships with serum markers of oxidation and inflammation. *HIV Med* 2010;11(4):225-31.
35. Falcone EL, Mangili A, Skinner S, Alam A, Polak JF, Wanke CA. Framingham risk score and early markers of atherosclerosis in a cohort of adults infected with HIV. *Antivir Ther* 2011;16(1):1-8.
36. Maggi P, Quirino T, Ricci E, De Socio GV, Gadaleta A, Ingrassia F, et al. Cardiovascular risk assessment in antiretroviral-naive HIV patients. *AIDS patient care and STDs* 2009;23(10):809-13.
37. D'Agostino RB, Sr., Grundy S, Sullivan LM, Wilson P, Group CHDRP. Validation of the Framingham coronary heart disease prediction scores: results of a multiple ethnic groups investigation. *Journal of the American Medical Association* 2001;286(2):180-87.
38. Brindle PM, McConnachie A, Upton MN, Hart CL, Davey Smith G, Watt GC. The accuracy of the Framingham risk-score in different socioeconomic groups: a prospective study. *Br J Gen Pract* 2005;55(520):838-45.

For peer review only - http://bmjopen.bmj.com/site/about/guidelines.xhtml

39. De Socio GV, Parruti G, Quirino T, Ricci E, Schillaci G, Adriani B, et al. Identifying HIV patients with an unfavorable cardiovascular risk profile in the clinical practice: results from the SIMONE study. *The Journal of infection* 2008;57(1):33-40.
40. Elzi L, Spoerl D, Voggensperger J, Nicca D, Simcock M, Bucher HC, et al. A smoking cessation programme in HIV-infected individuals: a pilot study. *Antivir Ther* 2006;11(6):787-95.

For peer review only

STROBE Statement—Checklist of items that should be included in reports of ***cohort studies***

	Item No	Recommendation
**Title and abstract**	1	(*a*) Indicate the study's design with a commonly used term in the title or the abstract Stated P1: Clinical Cohort Study
		(*b*) Provide in the abstract an informative and balanced summary of what was done and what was found – P2
**Introduction**		
Background/rationale	2	Explain the scientific background and rationale for the investigation being reported – P3
Objectives	3	State specific objectives, including any prespecified hypotheses – P3
**Methods**		
Study design	4	Present key elements of study design early in the paper – cohort description P4
Setting	5	Describe the setting, locations, and relevant dates, including periods of recruitment, exposure, follow-up, and data collection P4
Participants	6	(*a*) Give the eligibility criteria, and the sources and methods of selection of participants. Describe methods of follow-up P4
		(*b*) For matched studies, give matching criteria and number of exposed and unexposed - NA
Variables	7	Clearly define all outcomes, exposures, predictors, potential confounders, and effect modifiers. P4-5 Give diagnostic criteria, if applicable NA
Data sources/ measurement	8*	For each variable of interest, give sources of data and details of methods of assessment (measurement). Describe comparability of assessment methods if there is more than one group P4-5
Bias	9	Describe any efforts to address potential sources of bias P6 demographics of included and excluded patients were compared
Study size	10	Explain how the study size was arrived at - P6
Quantitative variables	11	Explain how quantitative variables were handled in the analyses. If applicable, describe which groupings were chosen and why P4-5
Statistical methods	12	(*a*) Describe all statistical methods, including those used to control for confounding P4-6
		(*b*) Describe any methods used to examine subgroups and interactions P6
		(*c*) Explain how missing data were addressed P4
		(*d*) If applicable, explain how loss to follow-up was addressed NA
		(*e*) Describe any sensitivity analyses P6
**Results**		
Participants	13*	(a) Report numbers of individuals at each stage of study—eg numbers potentially eligible, examined for eligibility, confirmed eligible, included in the study, completing follow-up, and analysed P6
		(b) Give reasons for non-participation at each stage P6
		(c) Consider use of a flow diagram
Descriptive data	14*	(a) Give characteristics of study participants (eg demographic, clinical, social) and information on exposures and potential confounders Table 1
		(b) Indicate number of participants with missing data for each variable of interest – we needed complete Framingham risk factors to assess heart age – restricted to all measured with 1 month P6
		(c) Summarise follow-up time (eg, average and total amount) NA we used latest available set of measurements P4

For peer review only - http://bmjopen.bmj.com/site/about/guidelines.xhtml

Outcome data	15*	Report numbers of outcome events or summary measures over time Table 1 and table 2
Main results	16	(*a*) Give unadjusted estimates and, if applicable, confounder-adjusted estimates and their precision (eg, 95% confidence interval). Make clear which confounders were adjusted for and why they were included – CI given throughout tables and results
		(*b*) Report category boundaries when continuous variables were categorized – tables 1-3
		(*c*) If relevant, consider translating estimates of relative risk into absolute risk for a meaningful time period – heart age is absolute
Other analyses	17	Report other analyses done—eg analyses of subgroups and interactions, and sensitivity analyses - P7-8
**Discussion**		
Key results	18	Summarise key results with reference to study objectives P8
Limitations	19	Discuss limitations of the study, taking into account sources of potential bias or imprecision. Discuss both direction and magnitude of any potential bias P8-9
Interpretation	20	Give a cautious overall interpretation of results considering objectives, limitations, multiplicity of analyses, results from similar studies, and other relevant evidence P10-12
Generalisability	21	Discuss the generalisability (external validity) of the study results P8
**Other information**		
Funding	22	Give the source of funding and the role of the funders for the present study and, if applicable, for the original study on which the present article is based – P15

*Give information separately for exposed and unexposed groups.

Note: An Explanation and Elaboration article discusses each checklist item and gives methodological background and published examples of transparent reporting. The STROBE checklist is best used in conjunction with this article (freely available on the Web sites of PLoS Medicine at http://www.plosmedicine.org/, Annals of Internal Medicine at http://www.annals.org/, and Epidemiology at http://www.epidem.com/). Information on the STROBE Initiative is available at http://www.strobe-statement.org.

For peer review only

For peer review only - http://bmjopen.bmj.com/site/about/guidelines.xhtml

Mind the gap: difference between Framingham heart age and real age increases with age in HIV-positive individuals: clinical cohort study

Journal:	*BMJ Open*
Manuscript ID:	bmjopen-2013-003245.R1
Article Type:	Research
Date Submitted by the Author:	20-Aug-2013
Complete List of Authors:	Davies, Teri-Louise; Bristol University, School of Social and Community Medicine<br>Gompels, Mark; North Bristol NHS Trust, HIV Service<br>Johnston, Sarah; North Bristol NHS Trust, HIV Service<br>Bovill, Begoña; North Bristol NHS Trust, HIV Service<br>May, Margaret; University of Bristol, School of Social and Community Medicine
**Primary Subject Heading**:	HIV/AIDS
Secondary Subject Heading:	Cardiovascular medicine
Keywords:	HIV & AIDS < INFECTIOUS DISEASES, Cardiac Epidemiology < CARDIOLOGY, EPIDEMIOLOGY

SCHOLARONE™ Manuscripts

For peer review only

Mind the gap: difference between Framingham heart age and real age increases with age in HIV-positive individuals: clinical cohort study

Teri-Louise Davies[1], Mark Gompels[2], Sarah Johnston[2], Begoña Bovill[2] and Margaret T. May[1]

[1] School of Social and Community Medicine, University of Bristol (MM Reader, TD PhD student)

[2] HIV Service, North Bristol NHS Trust, Bristol (MG Director and lead clinician, SJ and BB clinicians)

Running title: HIV heart age

Correspondence to:

Dr. Margaret May

Reader in Medical Statistics

School of Social and Community Medicine

University of Bristol

Bristol

BS8 2PS

UK

Telephone: +44 117 9287287

Fax: +44 117 928 7325

m.t.may@bristol.ac.uk

Key words: HIV, Cardiovascular disease, Framingham risk equation, heart, ageing

For peer review only

Abstract (275)

Objectives To measure the excess risk of cardiovascular disease (CVD) in HIV-positive individuals by comparing 'heart age' with real age and to estimate associations of patients characteristics with heart age deviation (heart age - real age).

Design Clinical Cohort Study

Setting Bristol HIV clinic, Brecon Unit at Southmead Hospital, Bristol, UK.

Participants 749 HIV-positive adults who attended for care between 2008-2011. Median age was 42 years (IQR 35-49), 67% were male and 82% were treated with antiretroviral therapy.

Main outcome measures We calculated the Framingham 10 year risk of CVD and traced back to 'heart age', the age of an individual with the same score but ideal risk factor values. We estimated the relationship between heart age deviation and real age using fractional polynomial regression. We estimated crude and mutually adjusted associations of sex, age, CD4 count, viral load/treatment status and period of starting antiretroviral therapy with heart age deviation.

Results The average heart age for a male aged 45 years was 48 years for a non-smoker and 60 years for a smoker. Heart age deviation increased with real age and at younger ages was smaller for females than males, although this reversed after age 48 years. Compared to patients with CD4 count <500 cells/mm^3, heart age deviation was 2.4 (95% CI 0.7-4.0) and 4.3 (2.3-6.3) years higher for those with CD4 500-749 cells/mm^3 and ≥750 cells/mm^3 respectively.

Conclusions In HIV-positive individuals, the difference between heart age and real age increased with age and CD4 count and was very dependent on smoking status. Heart age could be a useful tool to communicate CVD risk to patients and the benefits of stopping smoking.

For peer review only - http://bmjopen.bmj.com/site/about/guidelines.xhtml

Article summary

Article focus

- HIV-positive individuals may have a high lifetime risk of cardiovascular disease (CVD) because they are now living to much older ages and have a high prevalence of smoking.
- The Canadian Cardiovascular Society[1] has promoted the use of 'heart age', derived from the 10-year Framingham risk equations of general CVD, as a tool for communicating risk to the public and encouraging modification of risk factors.
- We estimated the difference between real age and 'heart age' in HIV positive individuals and investigated associations of clinical characteristics with higher heart age difference.

Key messages

- Our study of people in care for HIV infection in the UK showed that 'heart age' exceeds real age at all ages in men and above age 40 years old in women and is much higher in smokers.
- It is very important to estimate CVD risk in people who are HIV positive.
- Interventions on lifestyle factors such as smoking and obesity at young ages are required because the gap between 'heart age' and real age increases with age.

Strengths and limitations

For peer review only - http://bmjopen.bmj.com/site/about/guidelines.xhtml

For peer review only

- We were able to estimate Framingham risk and heart age in three-quarters of the patients in the Bristol Cohort.
- Some patient groups, such as those with a history of injection drug use, were under-represented and therefore our results may be more applicable to patients that regularly attend for HIV care.
- A major limitation of our study is that we did not have information on smoking status. We sought to overcome this by duplicating analyses assuming all were smokers and all were non-smokers.
- We lacked information on CVD events or deaths and so we do not know whether higher estimated CVD risk in this HIV population translates to an elevated rate of CVD.

Introduction

HIV-positive individuals are now living to much older ages [2,3] and therefore may be at high risk of cardiovascular disease (CVD). [4,5] Guidelines for the clinical management of HIV patients stress the importance of assessing risk of CVD and recommend interventions to treat risk factors.[6] Although there have been some attempts to introduce CVD risk scoring tools specifically for HIV-positive individuals,[7-9] none have been independently validated and therefore the Framingham risk equation[10] is still widely used,[11] particularly as the Framingham Heart Study website[12] provides a simple, accessible tool for calculating the risk of developing CVD within 10 years.

For peer review only - http://bmjopen.bmj.com/site/about/guidelines.xhtml

Communication of CVD risk to HIV patients is extremely important, particularly the impact of modifiable risk factors, such as smoking. Recently the Canadian Cardiovascular Society[1] promoted the use of 'heart age' derived from the 10-year Framingham risk equations for general CVD. [10] A person's 'heart age' is the age of an individual with the same risk score but ideal modifiable risk factor values. Therefore 'heart age' is a useful measure of excess CVD risk adjusted for age and sex.

Our objectives were to compare 'heart age' with actual age and to estimate the association of patient clinical and demographic characteristics with heart age deviation, the difference between estimated 'heart age' and their real age, in the Bristol Cohort of HIV-positive individuals.

For peer review only

Methods

Study participants

The Bristol HIV Cohort study enrols patients attending the Brecon Unit at Southmead Hospital, Bristol, UK. Routine clinical data collected on patients attending for HIV care up to November 2011were available for analysis as part of the UK CHIC study. In accordance with data protection policy, all data were anonymised. Included patients were aged 18 years and over and were not infected peri-natally.

Data measurement and availability

Demographic data on sex, date of birth, ethnicity (black African, white and other), assumed HIV transmission group and the dates of HIV diagnosis and first clinic visit were available. CD4 cell count and HIV-1 RNA were usually measured at each clinic visit. Details of antiretroviral therapy (ART) and non-HIV medications were available. Systolic blood pressure (SBP) and total and HDL cholesterol have been measured since 2008, at first visit and at least annually thereafter according to protocol. Patients ever recorded as taking anti-hypertensive medication were classed as treated for high blood pressure. Diagnosis of diabetes mellitus was recorded in patient notes. Patients with missing diabetes status were assumed to be non-diabetic. Smoking status was not available. Patients included in analyses had at least one set of Framingham risk factors (SBP, total and HDL cholesterol), CD4 count and HIV-1 RNA measured within a 6 month time window. We used the latest available measurements to calculate the Framingham risk score.

Statistical methods

Calculation of Framingham 10-year risk of CVD and heart age

For peer review only - http://bmjopen.bmj.com/site/about/guidelines.xhtml

We calculated the 10-year risk of CVD for each person using the sex-specific Framingham equations for general CVD[10 12] which include age, total and HDL cholesterol, SBP, treatment for hypertension, current smoking (yes/no) and diabetes status. We used the Framingham risk to trace back to 'heart age': the age of an individual with the same score but ideal risk factor values (non-smoker, non-diabetic, untreated SBP 125 mmHg, total cholesterol 180 mg/dL, HDL 45 mg/dL[10]). For example, if a 40 year old male has a 10-year CVD risk of 5.6%, his 'heart age' would be 45 years because a 45 year old male with ideal risk factors has a 10-year risk of CVD of 5.6%. For comparison, the 10-year CVD risk for a 40 year old male with ideal risk factors is 3.9%. Data on smoking status was not collected in the Bristol cohort and therefore analyses were conducted twice, firstly assuming all were smokers, and secondly assuming all non-smokers. Heart age deviation ('heart age' - real age) was calculated for each individual for each smoking assumption.

<u>Analysis of heart age deviation</u>

We estimated the difference between age and 'heart age' overall and by age group (18-39, 40-49, 50-59 and ≥ 60). We used box plots to compare the distribution of 'heart age' with median real age for male and female smokers and non-smokers stratified by real age group. We used fractional polynomial regression models[13] separately for male and female smokers and non-smokers to show the variation of heart age deviation with age. We used univariable and multivariable linear regression models to estimate crude and mutually adjusted associations of sex, age group, current CD4 count, treatment/viral load status (untreated, treated and suppressed, treated and not suppressed) and period of starting ART (pre v. post 2003) with heart age deviation. We also considered models that included duration since HIV diagnosis, duration since first clinic visit, duration and type of ART.

For peer review only

In sensitivity analyses, we repeated the main analyses firstly including only those on ART and secondly restricting to males. We also tested whether CD4 count was associated with total cholesterol, the ratio of total to HDL cholesterol and SBP, controlling for age and sex. Results are presented as the difference between heart age and real age in years with 95% confidence intervals. All calculations were executed in STATA version 12.1 [14].

Results

Of the 1,013 patients who attended the clinic, 749 (74%) had measurements of CD4 count, HIV-1 RNA, total and HDL cholesterol, SBP taken within a 6 month period. Patient demographic and clinical characteristics at the time of the Framingham risk assessment are shown in table 1. Two thirds of the patients were male, the majority of whom were men who have sex with men (MSM) and of white ethnicity. In contrast, the majority of females (63%) were of Black African origin. Compared to those included in analyses, excluded individuals (N=264) without risk factor measurements were similar in age, sex and ethnicity, but were twice as likely to be infected via injection drug use (IDU), blood product or "unknown" risk group. The median latest CD4 count was 484 (IQR 322-657) mm^3 in those excluded which was intermediate between the treated and untreated included patients (table 1).

Table 2 shows real age compared with heart age and estimated heart age deviation overall and by age group, stratified by sex and smoking status. Heart age was greater than real age for all groups except for non-smoking females aged 18-39 years. The mean age of the men was 44.3 years and their mean heart age was 47.7 years if a non-smoker or 59.2 years if a smoker. The females were slightly younger with a mean age of 40.6 years and corresponding mean heart ages of 42.4 and 53.1 years assuming non-smoker and smoker respectively.

For peer review only - http://bmjopen.bmj.com/site/about/guidelines.xhtml

Figure 1 illustrates the distribution of heart age by age group. The box represents the middle 50% of the distribution with the median marked as a line, and the tails extend to 95% of the distribution with outliers marked as dots. There was an increasing trend across age groups in the deviation between the median age and median heart age.

Figure 2 shows heart age deviation increased substantially with real age and was much higher for smokers eg for males aged 50, the heart age deviation was around 18 years in smokers and around 5 years in non-smokers (illustrated by green line). At younger ages, females had smaller heart age deviation than males, but this reversed after about age 48. However, the deviation is relative to sex-specific risk in those with ideal risk factors and females have lower absolute risk than males in the general population. In our study, prevalence of diabetes increased with age, as expected. Median total and HDL cholesterol were higher in men aged ≥ 40 years compared with younger men, and were also higher in women aged ≥ 50 years compared with younger women.

The crude and adjusted associations of variables with heart age deviation are shown in table 3. Duration since HIV diagnosis, duration since first clinic visit, and duration of ART were not associated with heart age deviation. Compared with ages 18-39, those aged ≥ 60 years had an increase of 8.87 (95% CI 5.90 to 11.84) years in heart age deviation, and this was approximately doubled in smokers. Compared with those with CD4 count <500 cells/mm^3, those with CD4 count ≥ 750 cells/mm^3 had an increase of 4.28 (95% CI 2.28 to 6.27) years in heart age deviation and this effect was independent of smoking status. When analyses were restricted to males only, the associations of age and CD4 count with heart age deviation were somewhat weaker. Patterns of results were similar when analyses were restricted to

For peer review only - http://bmjopen.bmj.com/site/about/guidelines.xhtml

individuals on ART. There was no evidence of a difference in heart age deviation between those on PI- compared with NNRTI-based ART at the time of the CVD risk assessment. Higher total cholesterol (adjusted for age and sex), was associated with higher CD4 count (80 mg/dL increase in cholesterol per 50 increase in CD4 count, p=0.01) and this was not attenuated by adjusting for HDL cholesterol. Higher total:HDL cholesterol was similarly associated with higher CD4 count. SBP was not associated with CD4 count. There was some evidence that treated patients with unsuppressed virus, but not untreated patients, had greater heart deviation than those with suppressed virus.

Discussion

Main results

We showed that in the Bristol HIV cohort on average 'heart age' was greater than real age for men of all ages and for women aged over 40 years old. Heart age deviation widened with increasing age and was very much higher if people smoke. On average, a 45 year-old male smoker had a 'heart age' of around 60 years. Our results suggest that in women the difference between age and 'heart age' increased steeply after menopausal age. Untreated patients and treated patients who were virally suppressed had similar heart ages, but those who were not virally suppressed on ART had higher heart age deviation. Higher CD4 count was associated with higher heart age deviation.

Strengths and limitations

As far as we are aware, this is the first study to calculate 'heart age' based on the Framingham CVD risk score for HIV-positive individuals. Complete data on Framingham risk factors and HIV biomarkers were only available on 74% of the patients in the Bristol

For peer review only - http://bmjopen.bmj.com/site/about/guidelines.xhtml

Cohort and some patient groups such as IDU were under-represented. Therefore our results may be more applicable to patients that regularly attend for HIV care. Measurement error and misclassification may have biased our results. We only used one cross-sectional assessment of Framingham CVD risk score and cholesterol measurements were not all fasting measures. We assumed that individuals without diabetes status recorded were not diabetic and that those who had ever been prescribed medication for high blood pressure remained treated which may have resulted in some misclassification. A major limitation of our study is that we did not have information on smoking status. We sought to overcome this by duplicating analyses assuming all were smokers and all were non-smokers. Our results may be biased if smoking is associated with changes in other risk factors such as SBP and cholesterol that are intermediate in the pathway from smoking to CVD. Our study was limited to cross-sectional analysis and therefore does not show within person changes in CVD risk. We do not yet have full information on CVD events or deaths and so we do not know whether higher estimated CVD risk in this HIV population translates to an elevated rate of CVD. A much larger number of patients would be required to properly analyse CVD events and causes of death.

The calculation of "heart age" uses a set of "ideal" risk factor values proposed by the Framingham investigators. It is likely that "normal" or "average" CVD risk factor values in the UK general population are somewhat worse than the "ideal". According to the Health Survey for England 2006 which reported CVD risk factors in adults, for males aged >35 years the mean total and HDL cholesterol were 220mg/dl and 50mg/dl respectively, somewhat higher than the ideal values of 180mg/dl and 45mg/dl, and only 69% had SBP<140 and DBP<90 mmHg without medication.[15] Unfortunately, we did not have an age and sex matched HIV negative population for direct comparison.

For peer review only - http://bmjopen.bmj.com/site/about/guidelines.xhtml

In context with other studies

Our finding that heart age was greater than real age for the majority of HIV-positive individuals in this study population is concordant with a study that found increased CVD rates among HIV-positive compared with negative controls.[16] The increased burden of CVD among HIV-positive individuals is likely a consequence of increased traditional risk factors, including dyslipidemia and insulin resistance, and non-traditional risk factors such as immune activation and inflammation that may contribute to the accelerated ageing process characterised by higher than expected rates of non-infectious co-morbidities.[17] Higher prevalence of smoking also contributes to the CVD epidemic in the HIV-positive population[18 19] as may the use of recreational drugs. ART itself may contribute to CVD, but no effect of current PI use was detected in this UK cohort.

We found that heart age deviation was greater at older ages. This is particularly significant as the mean age of HIV infection is increasing and it is predicted that by 2020 in the USA 50% of people living with HIV will be over 50 years old.[20] Although our study was cross-sectional, it is likely that the gap between real and heart age increases within individuals as they age. Older HIV-positive individuals, compared with matched controls, have been found to have a higher prevalence of hypertension, hypertriglyceridemia , low bone density and lipodystrophy suggesting that HIV and treatment related factors accelerate normal ageing.[4] However, in our study, the widening of the gap between heart age and real age seen in older patients must be entirely driven by the risk factors included in the Framingham equation, namely diabetes, SBP, total and HDL cholesterol, being worse at older ages.

Our finding that higher current CD4 count was associated with higher estimated Framingham risk of CVD is to be interpreted with caution since the SMART study of structured treatment

For peer review only - http://bmjopen.bmj.com/site/about/guidelines.xhtml

interruption based on CD4 count found that CVD events were greater in those with lower CD4.[21] However, our results are in line with another study that found a higher prevalence of clinically evident lipodystrophy and higher CD4 cell counts in patients with higher Framingham score.[22] A study that used cardiac computed tomography imaging to identify coronary artery calcium found that vascular age was increased in over 40% of patients, with an average increase of 15 years over the chronological age, also found that current CD4 count was associated with higher vascular age.[23] Atherosclerosis is an inflammatory process of the subintimal layer of the arterial wall in which lymphocytes and macrophages play a major role. The CD4+ type 1 T helper (Th1) lymphocyte is the predominant subtype of T cells in atherosclerotic plaques of humans. [24 25] Furthermore it has been demonstrated in a mouse model that CD4 cells play a pathogenic role in atherosclerosis. [26] Therefore ART-induced increase in CD4 count may contribute to the development of atherosclerosis in HIV-positive patients. Other studies have shown that low CD4 count (<350cell/ml) is associated with higher rates of CVD or subclinical atherosclerosis. It may be that the association of CD4 with CVD is a U-shaped curve with low CD4 associated with acute inflammatory processes and high CD4 associated with chronic ongoing inflammatory processes.[23] However, in our study the association of CD4 with heart age may be mediated through components of the Framingham risk score, such as SBP or cholesterol.

Risk prediction

Although some HIV-specific coronary heart disease (CHD) and CVD prediction algorithms have been proposed, [7 9] none have been externally validated by independent data and the Framingham risk score is still widely used.[11 27 28] Risk prediction in HIV-positive populations firstly focused on CHD,[8] but now the importance of risk assessment for CVD has

For peer review only - http://bmjopen.bmj.com/site/about/guidelines.xhtml

been recognised by guidelines.[6] D'Agostino summarised the state of CVD risk prediction as applied to HIV populations in a review article. [28] Studies in HIV populations have compared the degree of correlation of 3 traditional risk prediction algorithms, Framingham, Systematic Coronary Risk Evaluation (SCORE) and Prospective Cardiovascular Munster (PROCAM) equations.[29-31]. The estimation of relative effects of traditional risk factors on CVD outcomes appears similar between HIV-positive and HIV negative individuals.[32] However, it may be that HIV-specific risk equations that include HIV-specific risk factors may perform better than existing algorithms because of potential differences in etiology of CVD in the HIV population. For example, D-dimer, a marker of inflammation, has been found to be independently predictive of CVD events. [33] However, the Framingham risk score may partially capture inflammation since markers of inflammation have been found to be associated with a higher score in HIV patients compared with controls [34] and the score has been shown to correlate with the presence of subclinical atherosclerosis measured by carotid artery intima-media thickness in HIV-positive individuals.[35 36] Atherosclerosis may also be high in untreated patients, supporting a role of HIV infection itself as a risk factor.[37]

We used the Framingham risk score because it is based on readily available measures and widely used in clinical practice. Also, our focus was on factors that could be changed by lifestyle interventions, in particular smoking, blood pressure and cholesterol. Alternative scores have used different risk factors, for example, QRISK[38] includes ethnicity and family history of coronary heart disease which are not modifiable, body mass index (BMI), deprivation score, atrial fibrillation, rheumatoid arthritis, and chronic renal disease. Although these risk factors are not entered in the Framingham risk score, they may be important in the evaluation of clinical risk. For example, renal insufficiency has a relatively high prevalence

For peer review only - http://bmjopen.bmj.com/site/about/guidelines.xhtml

in HIV infected black patients and may be an important contributor to risk in this population.[39]

The ability to accurately predict CVD risk is an essential element of this population's care. The Framingham equation for CHD predicted well in The Data Collection on Adverse events of Anti-HIV Drugs Study (D:A:D) in terms of discrimination, but tended to underestimate risk in smokers.[8] The Framingham risk scores may require recalibration to adjust for over or under prediction in the HIV population.[40 41] Factors unique to HIV, such as effects of different antiretroviral drugs, may influence the performance of standard risk prediction tools, as they may change CVD risk both through alterations of traditional risk factors and by contributing to inflammatory and immunologic risk factors. D:A:D found both observed and predicted rates of MI increased with time on ART implying that ART-induced changes in conventional risk factors at least partially explained the increase in risk of MI.[8] The D:A:D predictive model for CVD tailored to HIV patients included exposure to indinavir, boosted lopinavir and abacavir as well as the traditional Framingham risk factors.[9]

The HIV-HEART study found that not only were CVD risk factors high in the HIV population, but also that they are under-treated and therefore risk factor management of HIV patients requires further improvement.[27] The European AIDS Clinical Society (EACS) guidelines on the prevention and management of metabolic disease in HIV [6] state that CVD risk should be assessed in HIV-positive individuals at regular intervals. They recommend lifestyle interventions should focus on counselling to stop smoking, modify diet and take regular exercise. A healthy diet, exercise and maintaining normal body weight tend to reduce dyslipidemia.

For peer review only - http://bmjopen.bmj.com/site/about/guidelines.xhtml

Smoking

Increased Framingham risk scores have been found in HIV-positive compared with negative controls, but this has mostly been due to higher prevalence of smoking rather than to higher cholesterol.[42] Smoking is the most important modifiable risk factor. A pilot study of a smoking cessation programme using counselling and nicotine replacement therapy in the Swiss HIV Cohort Study [43] found that implementing a smoking cessation programme was feasible in HIV-positive individuals. The D:A:D study found that the adjusted incidence rate ratio of CVD decreased from 2.32 within the first year of stopping smoking to 1.49 after greater than 3 years compared with those who never smoked. A recent study from Denmark estimated that HIV-positive individuals lose more years from smoking than from HIV infection.[19]

Future work

In order for accurate CVD risk assessment to be carried out, HIV cohorts need to collect better information on Framingham risk factors and, in particular, on current smoking status of patients. Our study should be considered as a pilot study for assessing heart age. Ideally, this type of analysis would need to be rolled out into a much larger national cohort to properly gauge which factors predict heart age deviation. In addition, CVD events and death would allow us to test whether the Framingham equations are transferable to the UK HIV population. We need to assess the use of heart age as a communication tool for behaviour intervention. More research is required to investigate whether Framingham risk estimates accurately translate to actual CVD events and deaths or whether including HIV specific risk

For peer review only - http://bmjopen.bmj.com/site/about/guidelines.xhtml

factors in the CVD risk equations might result in a more accurate prediction tool for this population.

Implications and conclusion

HIV-positive individuals in this cohort had a considerably increased risk of CVD compared with the ideal reference values. Our research, which showed that heart age exceeds real age at all ages in men and above age 40 years old in women, implies that it is important to estimate CVD risk in HIV-positive individuals. The effect of smoking is to increase heart age on average by 8 to 17 years in males and 8 to 18 years in females depending on the mean real age. This indicates the importance of smoking cessation for prevention of CVD in this population. Furthermore, since the gap between heart age and real age increased as people go older, it is important to intervene on lifestyle factors such as smoking and obesity at young ages. Cardiovascular risk expressed in terms of an individual's heart age, rather than absolute risk of CVD, may have more impact on patients in programmes aiming to intervene on risk factors. Heart age may be a particularly useful tool in communicating risk to younger patients who are at low absolute risk of CVD. Tracking change in heart age, such as that due to smoking cessation, may provide strong motivation towards life style changes.

For peer review only

For peer review only - http://bmjopen.bmj.com/site/about/guidelines.xhtml

Acknowledgements: We thank all patients, Susan Allan, and the team at Southmead Hospital, Bristol, UK. This work was presented in part at the Eleventh International Congress on Drug Therapy in HIV Infection Glasgow, UK 11-15 November 2012. Abstract available: Davies T et al. Journal of the International AIDS Society 2012, 15(Suppl 4):18152

http://www.jiasociety.org/index.php/jias/article/view/18152 | http://dx.doi.org/10.7448/IAS.15.6.18152

Contributors: MG and MM contributed to study design and interpretation of analyses. TD analysed the data, compiled the tables and graphs and wrote the first draft of the paper. MM supervised statistical analyses. All authors contributed to the writing of the paper, edited, revised and approved the final version of the paper and had full access to all the data in the study. MM had the final responsibility for the decision to submit for publication and is the guarantor.

Funding: Margaret May is funded by the Medical Research Council, UK (grant MR/J002380/1). Teri Davies is funded by the UK Medical Research Council as a PhD student in the Bristol Centre for Systems Biomedicine (BCSBmed) doctoral training centre. The views expressed in this manuscript are those of the researchers and not necessarily those of the Medical Research Council. The funders had no role in carrying out the study or in the decision to submit the manuscript for publication.

Competing interests: none.

Ethical approval: The project was approved by a Multi-centre Research Ethics Committee and by local ethics committees.

Copyright: The Corresponding Author has the right to grant on behalf of all authors and does grant on behalf of all authors, an exclusive licence (or non exclusive for government employees) on a worldwide basis to the BMJ Publishing Group Ltd to permit this article (if accepted) to be published in BMJ editions and any other BMJPGL products and sublicences such use and exploit all subsidiary rights, as set out in our licence.

For peer review only - http://bmjopen.bmj.com/site/about/guidelines.xhtml

Data sharing: no additional data available.

For peer review only

For peer review only - http://bmjopen.bmj.com/site/about/guidelines.xhtml

Table 1: Patient demographic and clinical characteristics at time of Framingham Score assessment

	Number of Patients N = 749	Percent
**Sex**		
Male	503	67.2
**Transmission risk group**		
Heterosexual	386	51.5
MSM	312	41.7
IDU	23	3.1
Blood Product	7	0.9
Unknown	21	2.8
**Ethnicity**		
Black African	228	30.4
White	441	58.9
Other	80	10.7
**Age (years) (median, IQR)**	42.2	(35.5-49.4)
**Age category (years)**		
18-39	310	41.4
40-49	265	35.4
50-59	118	15.8
60+	56	7.5
**Treatment status**		
On ART	612	81.7
**Started ART pre 01/01/2003**	168	22.4
**Viral load (copies per ml)**		
Treated and suppressed (vl $\leq$ 50)	535	71.4
Treated and unsuppressed	77	10.3
Untreated	137	18.3
**Median cd4 count (mm^3), untreated (IQR)**	477	(334-602)
**Median cd4 count (mm^3), treated (IQR)**	559.5	(409-721)
**cd4 count (mm^3)**		
<500	318	42.5
500 - 749	275	36.7
$\geq$ 750	156	20.8
**Median total cholesterol(mg/dL), (IQR)**	189.1	(162-220)
**Median HDL cholesterol(mg/dL), (IQR)**	50.2	(38.6-61.8)
**Median systolic blood pressure(mmHg), (IQR)**	131	(117-143)

For peer review only

For peer review only - http://bmjopen.bmj.com/site/about/guidelines.xhtml

HIV heart age

Table 2: Age, heart age and heart age deviation (heart age- real age) by age group for male and female smokers and non-smokers.

sex	smoking assumption	real age category (years)	n (%)	mean real age (years)	mean heart age (years)	deviation=heart age-real age (years) mean	(95% CI)
males	non-smoker	18-39	184(37%)	33.5	34.9	1.3	(0.60,2.09)
		40-49	182 (36%)	45.1	48.5	3.3	(2.17,4.53)
		50-59	91 (18%)	53.4	59.5	6.1	(3.85,8.37)
		60-	46 (9%)	66.2	73.0	6.8	(2.19,11.36)
		total	503(100%)	44.3	47.7	3.4	(2.65,4.21)
males	smoker	18-39	184(37%)	33.5	43.3	9.8	(8.78,10.74)
		40-49	182 (36%)	45.1	60.1	15.0	(13.53,16.48)
		50-59	91 (18%)	53.4	73.9	20.4	(17.64,23.24)
		60-	46 (9%)	66.2	89.6	23.3	(18.35,28.29)
		total	503(100%)	44.3	59.2	14.8	(13.82,15.84)
females	non-smoker	18-39	126(51%)	33.1	31.7	-1.4	(-2.93,0.05)
		40-49	83 (34%)	44.1	47.1	3.0	(-0.13,6.10)
		50-59	27 (11%)	55.2	62.9	7.7	(1.54,13.93)
		60-	10 (4%)	65.9	82.2	16.3	(0.07,32.52)
		total	246(100%)	40.6	42.4	1.8	(0.15,3.41)
females	smoker	18-39	126(51%)	33.1	39.9	6.8	(4.87,8.64)
		40-49	83 (34%)	44.1	59.2	15.0	(11.19,18.90)
		50-59	27 (11%)	55.2	78.9	23.7	(15.77,31.55)
		60-	10 (4%)	65.9	99.8	33.9	(16.79,51.01)
		total	246(100%)	40.6	53.1	12.5	(10.42,14.61)

For peer review only

For peer review only - http://bmjopen.bmj.com/site/about/guidelines.xhtml

HIV heart age

Table 3: Crude and mutually adjusted heart age deviation (difference from comparator group*) according to patient characteristics for non-smokers and smokers

	All individuals (N=749)			
	Non smoker Deviation (years) (95% CI)		Smoker Deviation (years) (95% CI)	
**Variable**	**Crude**	**Adjusted**	**Crude**	**Adjusted**
**Female vs Male**	-1.65 (-3.25,-0.05)	-0.96 (-2.52,0.59)	-2.32 (-4.38,-0.25)	-0.72(-2.62,1.18)
**Age category**	-	-	-	-
18-39*	-	-	-	-
40-49	3.02 (1.35,4.69)	3.38 (1.66,5.10)	6.48(4.44,8.52)	6.75(4.65,8.86)
50-59	6.27(4.11,8.43)	6.39(4.17,8.61)	12.64(10.00,15.27)	12.64(9.94,15.35)
60+	8.26(5.36,11.16)	8.87(5.90,11.84)	16.67(13.13,20.20)	17.21(13.59,20.83)
**CD4 count category (cells/mm^3)**	-	-	-	-
<500*	-	-	-	-
500-750	1.91(0.23,3.60)	2.39(0.74,4.03)	2.50(0.34,4.66)	3.01(1.00,5.02)
>=750	4.23(2.23,6.23)	4.28(2.28,6.27)	6.12(3.55,8.68)	5.44(3.01,7.87)
**Viral load category**	-	-	-	-
Treated with suppressed vl*	-	-	-	-
Treated with unsuppressed vl	1.07(-1.44,3.59)	2.84(0.40,5.29)	-0.19(-3.42,3.04)	2.90(-0.08,5.89)
Untreated	-1.39(-3.36,0.58)	0.27(-1.74,2.27)	-3.61(-6.15,-1.07)	-0.28(-2.73,2.17)
**ART start date pre 01/01/2003**	0.18(-1.62,1.99)	-2.28(-4.14,-0.42)	1.98(-0.35,4.30)	-2.43(-4.70,-0.17)
**Constant**	-	-1.26(-3.00,0.49)	-	6.70(4.57,8.82)

For peer review only - http://bmjopen.bmj.com/site/about/guidelines.xhtml

HIV heart age

Figure 1: Box plots of distribution of heart age by age group for female non-smokers (upper left), female smokers (upper right), male non-smokers (lower left), male smokers (lower right). The median real age for each age group is shown in red for comparison. In the box plot, the whiskers include 95% of the distribution and the box includes the middle 50% with the median marked as a line. Outliers are shown as filled circles.

For peer review only

HIV heart age

Figure 2: Heart age deviation in years (95% CI shown shaded in gray) for male and female smokers and non-smokers for real ages 20 to 70 years old

For peer review only

For peer review only - http://bmjopen.bmj.com/site/about/guidelines.xhtml

REFERENCES

1. Canadian Cardiovascular Society. CCS Guideline Programs: Updated CCS Dyslipidemia Guidelines Knowledge Translation Tools., 2013. http://www.ccsguidelineprograms.ca/index.php accessed 02/08/2013.
2. May MT, Ingle SM. Life expectancy of HIV-positive adults: a review. *Sex Health* 2011;8(4):526-33.
3. Nakagawa F, Lodwick RK, Smith CJ, et al. Projected life expectancy of people with HIV according to timing of diagnosis. *AIDS* 2012;26:335-43.
4. Onen NF, Overton ET, Seyfried W, et al. Aging and HIV infection: a comparison between older HIV-infected persons and the general population. *HIV clinical trials* 2010;11(2):100-9.
5. Law M, Friis-Moller N, Weber R, et al. Modelling the 3-year risk of myocardial infarction among participants in the Data Collection on Adverse Events of Anti-HIV Drugs (DAD) study. *HIV.Med* 2003;4(1):1-10.
6. Lundgren JD, Battegay M, Behrens G, et al. European AIDS Clinical Society (EACS) guidelines on the prevention and management of metabolic diseases in HIV. *HIV Med* 2008;9(2):72-81.
7. May M, Sterne JA, Shipley M, et al. A coronary heart disease risk model for predicting the effect of potent antiretroviral therapy in HIV-1 infected men. *International journal of epidemiology* 2007;36(6):1309-18.
8. Law MG, Friis-Moller N, El-Sadr WM, et al. The use of the Framingham equation to predict myocardial infarctions in HIV-infected patients: comparison with observed events in the D:A:D Study. *HIV.Med.* 2006;7(4):218-30.
9. Friis-Moller N, Thiebaut R, Reiss P, et al. Predicting the risk of cardiovascular disease in HIV-infected patients: the Data collection on Adverse Effects of Anti-HIV Drugs Study. *Eur J Cardiovasc Prev Rehabil.* 2010.
10. D'Agostino RB, Sr., Vasan RS, Pencina MJ, et al. General cardiovascular risk profile for use in primary care: the Framingham Heart Study. *Circulation* 2008;117(6):743-53.
11. Burke EG, Nelson J, Kwong J, et al. Cardiovascular risk assessment for persons living with HIV. *The Journal of the Association of Nurses in AIDS Care : JANAC* 2012;23(2):134-45.
12. Framingham Heart Study. General Cardiovascular Disease (10-year risk), 2011 http://www.framinghamheartstudy.org/risk/gencardio.html Accessed 02/08/2013
13. Royston P, Ambler G, Sauerbrei W. The use of fractional polynomials to model continuous risk variables in epidemiology. *Int.J.Epidemiol.* 1999;28(5):964-74.
14. Stata Statistical Software: Release 12 [program]. College Station, TX: StataCorp LP., 2011.
15. Health Survey for England - 2006, CVD and risk factors for adults, obesity and risk factors for children 2008. Available from: www.ic.nhs.uk/pubs/HSE06CVDandriskfactors. accessed 15/01/2013
16. Triant VA, Lee H, Hadigan C, et al. Increased acute myocardial infarction rates and cardiovascular risk factors among patients with human immunodeficiency virus disease. *The Journal of clinical endocrinology and metabolism* 2007;92(7):2506-12.
17. Petoumenos K, Worm SW. HIV infection, aging and cardiovascular disease: epidemiology and prevention. *Sex Health* 2011;8(4):465-73.
18. Smith CJ, Levy I, Sabin CA, et al. Cardiovascular disease risk factors and antiretroviral therapy in an HIV-positive UK population. *HIV Med* 2004;5(2):88-92.
19. Helleberg M, Afzal S, Kronborg G, et al. Mortality Attributable to Smoking Among HIV-1-Infected Individuals: A Nationwide, Population-Based Cohort Study. *Clin Infect Dis* 2013;56(5):727-34.
20. Neergaard L. Aging AIDS epidemic raises new questions. *NBC News*, 2012. Available from: http://www.nbcnews.com/id/48346557/ns/health-aids/#.USd556XvioZ. accessed 15/01/2013

21. El-Sadr WM, Lundgren JD, Neaton JD, et al. CD4+ count-guided interruption of antiretroviral treatment. *N.Engl.J.Med.* 2006;355(22):2283-96.

For peer review only - http://bmjopen.bmj.com/site/about/guidelines.xhtml

22. De Socio GV, Martinelli C, Ricci E, et al. Relations between cardiovascular risk estimates and subclinical atherosclerosis in naive HIV patients: results from the HERMES study. *Int J STD AIDS* 2010;21(4):267-72.
23. Guaraldi G, Zona S, Alexopoulos N, et al. Coronary aging in HIV-infected patients. *Clin Infect Dis* 2009;49(11):1756-62.
24. Frostegard J, Ulfgren AK, Nyberg P, et al. Cytokine expression in advanced human atherosclerotic plaques: dominance of pro-inflammatory (Th1) and macrophage-stimulating cytokines. *Atherosclerosis* 1999;145(1):33-43.
25. Maggi P, Perilli F, Lillo A, et al. An ultrasound-based comparative study on carotid plaques in HIV-positive patients vs. atherosclerotic and arteritis patients: atherosclerotic or inflammatory lesions? *Coronary artery disease* 2007;18(1):23-9.
26. Zhou X, Nicoletti A, Elhage R, et al. Transfer of CD4(+) T cells aggravates atherosclerosis in immunodeficient apolipoprotein E knockout mice. *Circulation* 2000;102(24):2919-22.
27. Reinsch N, Neuhaus K, Esser S, et al. Are HIV patients undertreated? Cardiovascular risk factors in HIV: results of the HIV-HEART study. *European journal of preventive cardiology* 2012;19(2):267-74.
28. D'Agostino RB, Sr. Cardiovascular risk estimation in 2012: lessons learned and applicability to the HIV population. *The Journal of infectious diseases* 2012;205 Suppl 3:S362-7.
29. Knobel H, Jerico C, Montero M, et al. Global cardiovascular risk in patients with HIV infection: concordance and differences in estimates according to three risk equations (Framingham, SCORE, and PROCAM). *AIDS patient care and STDs* 2007;21(7):452-7.
30. Guimaraes MMM, Greco DB, Garces AHI, et al. Coronary heart disease risk assessment in HIV-infected patients: a comparison of Framingham, PROCAM and SCORE risk assessment functions. *Int J Clin Pract* 2010;64(6):739-45.
31. Barros ZM, de Alencar Ximenes RA, Miranda-Filho DB, et al. Comparison between the Framingham and prospective cardiovascular of Munster scores for risk assessment of coronary heart disease in human immunodeficiency virus-positive patients in Pernambuco, Brazil. *Metabolic syndrome and related disorders* 2010;8(6):489-97.
32. Schambelan M, Wilson PW, Yarasheski KE, et al. Development of appropriate coronary heart disease risk prediction models in HIV-infected patients. *Circulation* 2008;118(2):e48-53.
33. Ford ES, Greenwald JH, Richterman AG, et al. Traditional risk factors and D-dimer predict incident cardiovascular disease events in chronic HIV infection. *AIDS* 2010;24(10):1509-17.
34. Guzman-Fulgencio M, Medrano J, Rallon N, et al. Soluble markers of inflammation are associated with Framingham scores in HIV-infected patients on suppressive antiretroviral therapy. *J Infection* 2011;63(5):382-90.
35. Parra S, Coll B, Aragones G, et al. Nonconcordance between subclinical atherosclerosis and the calculated Framingham risk score in HIV-infected patients: relationships with serum markers of oxidation and inflammation. *HIV Med* 2010;11(4):225-31.
36. Falcone EL, Mangili A, Skinner S, et al. Framingham risk score and early markers of atherosclerosis in a cohort of adults infected with HIV. *Antivir Ther* 2011;16(1):1-8.
37. Maggi P, Quirino T, Ricci E, et al. Cardiovascular risk assessment in antiretroviral-naive HIV patients. *AIDS patient care and STDs* 2009;23(10):809-13.
38. Hippisley-Cox J, Coupland C, Vinogradova Y, et al. Predicting cardiovascular risk in England and Wales: prospective derivation and validation of QRISK2. *BMJ.* 2008;336(7659):1475-+.
39. Campbell LJ, Desai M, Hegazi A, et al. Renal Impairment Is Associated With Coronary Heart Disease in HIV-Positive Men. *HIV clinical trials* 2012;13(6):343-49.
40. D'Agostino RB, Sr., Grundy S, Sullivan LM, et al. Validation of the Framingham coronary heart disease prediction scores: results of a multiple ethnic groups investigation. *Journal of the American Medical Association* 2001;286(2):180-87.
41. Brindle PM, McConnachie A, Upton MN, et al. The accuracy of the Framingham risk-score in different socioeconomic groups: a prospective study. *Br J Gen Pract* 2005;55(520):838-45.

For peer review only - http://bmjopen.bmj.com/site/about/guidelines.xhtml

42. De Socio GV, Parruti G, Quirino T, et al. Identifying HIV patients with an unfavorable cardiovascular risk profile in the clinical practice: results from the SIMONE study. *The Journal of infection* 2008;57(1):33-40.
43. Elzi L, Spoerl D, Voggensperger J, et al. A smoking cessation programme in HIV-infected individuals: a pilot study. *Antivir Ther* 2006;11(6):787-95.

For peer review only

For peer review only - http://bmjopen.bmj.com/site/about/guidelines.xhtml

STROBE Statement—Checklist of items that should be included in reports of ***cohort studies***

	Item No	Recommendation
**Title and abstract**	1	(*a*) Indicate the study's design with a commonly used term in the title or the abstract Stated P1: Clinical Cohort Study
		(*b*) Provide in the abstract an informative and balanced summary of what was done and what was found – P2
**Introduction**		
Background/rationale	2	Explain the scientific background and rationale for the investigation being reported – P3
Objectives	3	State specific objectives, including any prespecified hypotheses – P3
**Methods**		
Study design	4	Present key elements of study design early in the paper – cohort description P4
Setting	5	Describe the setting, locations, and relevant dates, including periods of recruitment, exposure, follow-up, and data collection P4
Participants	6	(*a*) Give the eligibility criteria, and the sources and methods of selection of participants. Describe methods of follow-up P4
		(*b*) For matched studies, give matching criteria and number of exposed and unexposed - NA
Variables	7	Clearly define all outcomes, exposures, predictors, potential confounders, and effect modifiers. P4-5 Give diagnostic criteria, if applicable NA
Data sources/ measurement	8*	For each variable of interest, give sources of data and details of methods of assessment (measurement). Describe comparability of assessment methods if there is more than one group P4-5
Bias	9	Describe any efforts to address potential sources of bias P6 demographics of included and excluded patients were compared
Study size	10	Explain how the study size was arrived at - P6
Quantitative variables	11	Explain how quantitative variables were handled in the analyses. If applicable, describe which groupings were chosen and why P4-5
Statistical methods	12	(*a*) Describe all statistical methods, including those used to control for confounding P4-6
		(*b*) Describe any methods used to examine subgroups and interactions P6
		(*c*) Explain how missing data were addressed P4
		(*d*) If applicable, explain how loss to follow-up was addressed NA
		(*e*) Describe any sensitivity analyses P6
**Results**		
Participants	13*	(a) Report numbers of individuals at each stage of study—eg numbers potentially eligible, examined for eligibility, confirmed eligible, included in the study, completing follow-up, and analysed P6
		(b) Give reasons for non-participation at each stage P6
		(c) Consider use of a flow diagram
Descriptive data	14*	(a) Give characteristics of study participants (eg demographic, clinical, social) and information on exposures and potential confounders Table 1
		(b) Indicate number of participants with missing data for each variable of interest – we needed complete Framingham risk factors to assess heart age – restricted to all measured with 1 month P6
		(c) Summarise follow-up time (eg, average and total amount) NA we used latest available set of measurements P4

For peer review only

Outcome data	15*	Report numbers of outcome events or summary measures over time Table 1 and table 2
Main results	16	(*a*) Give unadjusted estimates and, if applicable, confounder-adjusted estimates and their precision (eg, 95% confidence interval). Make clear which confounders were adjusted for and why they were included – CI given throughout tables and results
		(*b*) Report category boundaries when continuous variables were categorized – tables 1-3
		(*c*) If relevant, consider translating estimates of relative risk into absolute risk for a meaningful time period – heart age is absolute
Other analyses	17	Report other analyses done—eg analyses of subgroups and interactions, and sensitivity analyses - P7-8
**Discussion**		
Key results	18	Summarise key results with reference to study objectives P8
Limitations	19	Discuss limitations of the study, taking into account sources of potential bias or imprecision. Discuss both direction and magnitude of any potential bias P8-9
Interpretation	20	Give a cautious overall interpretation of results considering objectives, limitations, multiplicity of analyses, results from similar studies, and other relevant evidence P10-12
Generalisability	21	Discuss the generalisability (external validity) of the study results P8
**Other information**		
Funding	22	Give the source of funding and the role of the funders for the present study and, if applicable, for the original study on which the present article is based – P15

*Give information separately for exposed and unexposed groups.

Note: An Explanation and Elaboration article discusses each checklist item and gives methodological background and published examples of transparent reporting. The STROBE checklist is best used in conjunction with this article (freely available on the Web sites of PLoS Medicine at http://www.plosmedicine.org/, Annals of Internal Medicine at http://www.annals.org/, and Epidemiology at http://www.epidem.com/). Information on the STROBE Initiative is available at http://www.strobe-statement.org.

For peer review only

For peer review only - http://bmjopen.bmj.com/site/about/guidelines.xhtml

Mind the gap: difference between Framingham heart age and real age increases with age in HIV-positive individuals: clinical cohort study

Teri-Louise Davies[1], Mark Gompels[2], Sarah Johnston[2], Begoña Bovill[2] and Margaret T. May[1]

[1] School of Social and Community Medicine, University of Bristol (MM Reader, TD PhD student)

[2] HIV Service, North Bristol NHS Trust, Bristol (MG Director and lead clinician, SJ and BB clinicians)

Running title: HIV heart age

Correspondence to:

Dr. Margaret May

Reader in Medical Statistics

School of Social and Community Medicine

University of Bristol

Bristol

BS8 2PS

UK

Telephone: +44 117 9287287

Fax: +44 117 928 7325

m.t.may@bristol.ac.uk

Key words: HIV, Cardiovascular disease, Framingham risk equation, heart, ageing

For peer review only - http://bmjopen.bmj.com/site/about/guidelines.xhtml

Abstract (275)

Objectives To measure the excess risk of cardiovascular disease (CVD) in HIV-positive individuals by comparing 'heart age' with real age and to estimate associations of patients characteristics with heart age deviation (heart age - real age).

Design Clinical Cohort Study

Setting Bristol HIV clinic, Brecon Unit at Southmead Hospital, Bristol, UK.

Participants 749 HIV-positive adults who attended for care between 2008-2011. Median age was 42 years (IQR 35-49), 67% were male and 82% were treated with antiretroviral therapy.

Main outcome measures We calculated the Framingham 10 year risk of CVD and traced back to 'heart age', the age of an individual with the same score but ideal risk factor values. We estimated the relationship between heart age deviation and real age using fractional polynomial regression. We estimated crude and mutually adjusted associations of sex, age, CD4 count, viral load/treatment status and period of starting antiretroviral therapy with heart age deviation.

Results The average heart age for a male aged 45 years was 48 years for a non-smoker and 60 years for a smoker. Heart age deviation increased with real age and at younger ages was smaller for females than males, although this reversed after age 48 years. Compared to patients with CD4 count <500 cells/mm^3, heart age deviation was 2.4 (95% CI 0.7-4.0) and 4.3 (2.3-6.3) years higher for those with CD4 500-749 cells/mm^3 and ≥750 cells/mm^3 respectively.

Conclusions In HIV-positive individuals, the difference between heart age and real age increased with age and CD4 count and was very dependent on smoking status. Heart age could be a useful tool to communicate CVD risk to patients and the benefits of stopping smoking.

For peer review only - http://bmjopen.bmj.com/site/about/guidelines.xhtml

Article summary

Article focus

- HIV-positive individuals may have a high lifetime risk of cardiovascular disease (CVD) because they are now living to much older ages and have a high prevalence of smoking.
- The Canadian Cardiovascular Society[1] has promoted the use of 'heart age', derived from the 10-year Framingham risk equations of general CVD, as a tool for communicating risk to the public and encouraging modification of risk factors.
- We estimated the difference between real age and 'heart age' in HIV positive individuals and investigated associations of clinical characteristics with higher heart age difference.

Key messages

- Our study of people in care for HIV infection in the UK showed that 'heart age' exceeds real age at all ages in men and above age 40 years old in women and is much higher in smokers.
- It is very important to estimate CVD risk in people who are HIV positive.
- Interventions on lifestyle factors such as smoking and obesity at young ages are required because the gap between 'heart age' and real age increases with age.

Strengths and limitations

For peer review only - http://bmjopen.bmj.com/site/about/guidelines.xhtml

- We were able to estimate Framingham risk and heart age in three-quarters of the patients in the Bristol Cohort.
- Some patient groups, such as those with a history of injection drug use, were under-represented and therefore our results may be more applicable to patients that regularly attend for HIV care.
- A major limitation of our study is that we did not have information on smoking status. We sought to overcome this by duplicating analyses assuming all were smokers and all were non-smokers.
- We lacked information on CVD events or deaths and so we do not know whether higher estimated CVD risk in this HIV population translates to an elevated rate of CVD.

Introduction

HIV-positive individuals are now living to much older ages [2 3] and therefore may be at high risk of cardiovascular disease (CVD). [4 5] Guidelines for the clinical management of HIV patients stress the importance of assessing risk of CVD and recommend interventions to treat risk factors.[6] Although there have been some attempts to introduce CVD risk scoring tools specifically for HIV-positive individuals,[7-9] none have been independently validated and therefore the Framingham risk equation[10] is still widely used,[11] particularly as the Framingham Heart Study website[12] provides a simple, accessible tool for calculating the risk of developing CVD within 10 years.

For peer review only

Communication of CVD risk to HIV patients is extremely important, particularly the impact of modifiable risk factors, such as smoking. Recently the Canadian Cardiovascular Society[1] promoted the use of 'heart age' derived from the 10-year Framingham risk equations for general CVD. [10] A person's 'heart age' is the age of an individual with the same risk score but ideal modifiable risk factor values. Therefore 'heart age' is a useful measure of excess CVD risk adjusted for age and sex.

Our objectives were to compare 'heart age' with actual age and to estimate the association of patient clinical and demographic characteristics with heart age deviation, the difference between estimated 'heart age' and their real age, in the Bristol Cohort of HIV-positive individuals.

For peer review only

Methods

Study participants

The Bristol HIV Cohort study enrols patients attending the Brecon Unit at Southmead Hospital, Bristol, UK. Routine clinical data collected on patients attending for HIV care up to November 2011were available for analysis as part of the UK CHIC study. In accordance with data protection policy, all data were anonymised. Included patients were aged 18 years and over and were not infected peri-natally.

Data measurement and availability

Demographic data on sex, date of birth, ethnicity (black African, white and other), assumed HIV transmission group and the dates of HIV diagnosis and first clinic visit were available. CD4 cell count and HIV-1 RNA were usually measured at each clinic visit. Details of antiretroviral therapy (ART) and non-HIV medications were available. Systolic blood pressure (SBP) and total and HDL cholesterol have been measured since 2008, at first visit and at least annually thereafter according to protocol. Patients ever recorded as taking anti-hypertensive medication were classed as treated for high blood pressure. Diagnosis of diabetes mellitus was recorded in patient notes. Patients with missing diabetes status were assumed to be non-diabetic. Smoking status was not available. Patients included in analyses had at least one set of Framingham risk factors (SBP, total and HDL cholesterol), CD4 count and HIV-1 RNA measured within a 6 month time window. We used the latest available measurements to calculate the Framingham risk score.

Statistical methods

Calculation of Framingham 10-year risk of CVD and heart age

For peer review only - http://bmjopen.bmj.com/site/about/guidelines.xhtml

We calculated the 10-year risk of CVD for each person using the sex-specific Framingham equations for general CVD[10 12] which include age, total and HDL cholesterol, SBP, treatment for hypertension, current smoking (yes/no) and diabetes status. We used the Framingham risk to trace back to 'heart age': the age of an individual with the same score but ideal risk factor values (non-smoker, non-diabetic, untreated SBP 125 mmHg, total cholesterol 180 mg/dL, HDL 45 mg/dL[10]). For example, if a 40 year old male has a 10-year CVD risk of 5.6%, his 'heart age' would be 45 years because a 45 year old male with ideal risk factors has a 10-year risk of CVD of 5.6%. For comparison, the 10-year CVD risk for a 40 year old male with ideal risk factors is 3.9%. Data on smoking status was not collected in the Bristol cohort and therefore analyses were conducted twice, firstly assuming all were smokers, and secondly assuming all non-smokers. Heart age deviation ('heart age' - real age) was calculated for each individual for each smoking assumption.

For peer review only

Analysis of heart age deviation

We estimated the difference between age and 'heart age' overall and by age group (18-39, 40-49, 50-59 and ≥ 60). We used box plots to compare the distribution of 'heart age' with median real age for male and female smokers and non-smokers stratified by real age group. We used fractional polynomial regression models[13] separately for male and female smokers and non-smokers to show the variation of heart age deviation with age. We used univariable and multivariable linear regression models to estimate crude and mutually adjusted associations of sex, age group, current CD4 count, treatment/viral load status (untreated, treated and suppressed, treated and not suppressed) and period of starting ART (pre v. post 2003) with heart age deviation. We also considered models that included duration since HIV diagnosis, duration since first clinic visit, duration and type of ART.

For peer review only - http://bmjopen.bmj.com/site/about/guidelines.xhtml

In sensitivity analyses, we repeated the main analyses firstly including only those on ART and secondly restricting to males. We also tested whether CD4 count was associated with total cholesterol, the ratio of total to HDL cholesterol and SBP, controlling for age and sex. Results are presented as the difference between heart age and real age in years with 95% confidence intervals. All calculations were executed in STATA version 12.1 [14].

Results

Of the 1,013 patients who attended the clinic, 749 (74%) had measurements of CD4 count, HIV-1 RNA, total and HDL cholesterol, SBP taken within a 6 month period. Patient demographic and clinical characteristics at the time of the Framingham risk assessment are shown in table 1. Two thirds of the patients were male, the majority of whom were men who have sex with men (MSM) and of white ethnicity. In contrast, the majority of females (63%) were of Black African origin. Compared to those included in analyses, excluded individuals (N=264) without risk factor measurements were similar in age, sex and ethnicity, but were twice as likely to be infected via injection drug use (IDU), blood product or "unknown" risk group. The median latest CD4 count was 484 (IQR 322-657) mm^3 in those excluded which was intermediate between the treated and untreated included patients (table 1).

Table 2 shows real age compared with heart age and estimated heart age deviation overall and by age group, stratified by sex and smoking status. Heart age was greater than real age for all groups except for non-smoking females aged 18-39 years. The mean age of the men was 44.3 years and their mean heart age was 47.7 years if a non-smoker or 59.2 years if a smoker. The females were slightly younger with a mean age of 40.6 years and corresponding mean heart ages of 42.4 and 53.1 years assuming non-smoker and smoker respectively.

For peer review only - http://bmjopen.bmj.com/site/about/guidelines.xhtml

Figure 1 illustrates the distribution of heart age by age group. The box represents the middle 50% of the distribution with the median marked as a line, and the tails extend to 95% of the distribution with outliers marked as dots. There was an increasing trend across age groups in the deviation between the median age and median heart age.

Figure 2 shows heart age deviation increased substantially with real age and was much higher for smokers eg for males aged 50, the heart age deviation was around 18 years in smokers and around 5 years in non-smokers (illustrated by green line). At younger ages, females had smaller heart age deviation than males, but this reversed after about age 48. However, the deviation is relative to sex-specific risk in those with ideal risk factors and females have lower absolute risk than males in the general population. In our study, prevalence of diabetes increased with age, as expected. Median total and HDL cholesterol were higher in men aged ≥ 40 years compared with younger men, and were also higher in women aged ≥ 50 years compared with younger women.

The crude and adjusted associations of variables with heart age deviation are shown in table 3. Duration since HIV diagnosis, duration since first clinic visit, and duration of ART were not associated with heart age deviation. Compared with ages 18-39, those aged ≥ 60 years had an increase of 8.87 (95% CI 5.90 to 11.84) years in heart age deviation, and this was approximately doubled in smokers. Compared with those with CD4 count <500 cells/mm^3, those with CD4 count ≥ 750 cells/mm^3 had an increase of 4.28 (95% CI 2.28 to 6.27) years in heart age deviation and this effect was independent of smoking status. When analyses were restricted to males only, the associations of age and CD4 count with heart age deviation were somewhat weaker. Patterns of results were similar when analyses were restricted to

For peer review only - http://bmjopen.bmj.com/site/about/guidelines.xhtml

individuals on ART. There was no evidence of a difference in heart age deviation between those on PI- compared with NNRTI-based ART at the time of the CVD risk assessment. Higher total cholesterol (adjusted for age and sex), was associated with higher CD4 count (80 mg/dL increase in cholesterol per 50 increase in CD4 count, p=0.01) and this was not attenuated by adjusting for HDL cholesterol. Higher total:HDL cholesterol was similarly associated with higher CD4 count. SBP was not associated with CD4 count. There was some evidence that treated patients with unsuppressed virus, but not untreated patients, had greater heart deviation than those with suppressed virus.

Discussion

Main results

We showed that in the Bristol HIV cohort on average 'heart age' was greater than real age for men of all ages and for women aged over 40 years old. Heart age deviation widened with increasing age and was very much higher if people smoke. On average, a 45 year-old male smoker had a 'heart age' of around 60 years. Our results suggest that in women the difference between age and 'heart age' increased steeply after menopausal age. Untreated patients and treated patients who were virally suppressed had similar heart ages, but those who were not virally suppressed on ART had higher heart age deviation. Higher CD4 count was associated with higher heart age deviation.

Strengths and limitations

As far as we are aware, this is the first study to calculate 'heart age' based on the Framingham CVD risk score for HIV-positive individuals. Complete data on Framingham risk factors and HIV biomarkers were only available on 74% of the patients in the Bristol

For peer review only - http://bmjopen.bmj.com/site/about/guidelines.xhtml

Cohort and some patient groups such as IDU were under-represented. Therefore our results may be more applicable to patients that regularly attend for HIV care. Measurement error and misclassification may have biased our results. We only used one cross-sectional assessment of Framingham CVD risk score and cholesterol measurements were not all fasting measures. We assumed that individuals without diabetes status recorded were not diabetic and that those who had ever been prescribed medication for high blood pressure remained treated which may have resulted in some misclassification. A major limitation of our study is that we did not have information on smoking status. We sought to overcome this by duplicating analyses assuming all were smokers and all were non-smokers. Our results may be biased if smoking is associated with changes in other risk factors such as SBP and cholesterol that are intermediate in the pathway from smoking to CVD. Our study was limited to cross-sectional analysis and therefore does not show within person changes in CVD risk. We do not yet have full information on CVD events or deaths and so we do not know whether higher estimated CVD risk in this HIV population translates to an elevated rate of CVD. A much larger number of patients would be required to properly analyse CVD events and causes of death.

The calculation of "heart age" uses a set of "ideal" risk factor values proposed by the Framingham investigators. It is likely that "normal" or "average" CVD risk factor values in the UK general population are somewhat worse than the "ideal". According to the Health Survey for England 2006 which reported CVD risk factors in adults, for males aged >35 years the mean total and HDL cholesterol were 220mg/dl and 50mg/dl respectively, somewhat higher than the ideal values of 180mg/dl and 45mg/dl, and only 69% had SBP<140 and DBP<90 mmHg without medication.[15] Unfortunately, we did not have an age and sex matched HIV negative population for direct comparison.

For peer review only - http://bmjopen.bmj.com/site/about/guidelines.xhtml

In context with other studies

Our finding that heart age was greater than real age for the majority of HIV-positive individuals in this study population is concordant with a study that found increased CVD rates among HIV-positive compared with negative controls.[16] The increased burden of CVD among HIV-positive individuals is likely a consequence of increased traditional risk factors, including dyslipidemia and insulin resistance, and non-traditional risk factors such as immune activation and inflammation that may contribute to the accelerated ageing process characterised by higher than expected rates of non-infectious co-morbidities.[17] Higher prevalence of smoking also contributes to the CVD epidemic in the HIV-positive population[18 19] as may the use of recreational drugs. ART itself may contribute to CVD, but no effect of current PI use was detected in this UK cohort.

We found that heart age deviation was greater at older ages. This is particularly significant as the mean age of HIV infection is increasing and it is predicted that by 2020 in the USA 50% of people living with HIV will be over 50 years old.[20] Although our study was cross-sectional, it is likely that the gap between real and heart age increases within individuals as they age. Older HIV-positive individuals, compared with matched controls, have been found to have a higher prevalence of hypertension, hypertriglyceridemia , low bone density and lipodystrophy suggesting that HIV and treatment related factors accelerate normal ageing.[4] However, in our study, the widening of the gap between heart age and real age seen in older patients must be entirely driven by the risk factors included in the Framingham equation, namely diabetes, SBP, total and HDL cholesterol, being worse at older ages.

Our finding that higher current CD4 count was associated with higher estimated Framingham risk of CVD is to be interpreted with caution since the SMART study of structured treatment

For peer review only - http://bmjopen.bmj.com/site/about/guidelines.xhtml

interruption based on CD4 count found that CVD events were greater in those with lower CD4.[21] However, our results are in line with another study that found a higher prevalence of clinically evident lipodystrophy and higher CD4 cell counts in patients with higher Framingham score.[22] A study that used cardiac computed tomography imaging to identify coronary artery calcium found that vascular age was increased in over 40% of patients, with an average increase of 15 years over the chronological age, also found that current CD4 count was associated with higher vascular age.[23] Atherosclerosis is an inflammatory process of the subintimal layer of the arterial wall in which lymphocytes and macrophages play a major role. The CD4+ type 1 T helper (Th1) lymphocyte is the predominant subtype of T cells in atherosclerotic plaques of humans. [24 25] Furthermore it has been demonstrated in a mouse model that CD4 cells play a pathogenic role in atherosclerosis. [26] Therefore ART-induced increase in CD4 count may contribute to the development of atherosclerosis in HIV-positive patients. Other studies have shown that low CD4 count (<350cell/ml) is associated with higher rates of CVD or subclinical atherosclerosis. It may be that the association of CD4 with CVD is a U-shaped curve with low CD4 associated with acute inflammatory processes and high CD4 associated with chronic ongoing inflammatory processes.[23] However, in our study the association of CD4 with heart age may be mediated through components of the Framingham risk score, such as SBP or cholesterol.

Risk prediction

Although some HIV-specific coronary heart disease (CHD) and CVD prediction algorithms have been proposed, [7 9] none have been externally validated by independent data and the Framingham risk score is still widely used.[11 27 28] Risk prediction in HIV-positive populations firstly focused on CHD,[8] but now the importance of risk assessment for CVD has

For peer review only - http://bmjopen.bmj.com/site/about/guidelines.xhtml

been recognised by guidelines.[6] D'Agostino summarised the state of CVD risk prediction as applied to HIV populations in a review article. [28] Studies in HIV populations have compared the degree of correlation of 3 traditional risk prediction algorithms, Framingham, Systematic Coronary Risk Evaluation (SCORE) and Prospective Cardiovascular Munster (PROCAM) equations.[29-31]. The estimation of relative effects of traditional risk factors on CVD outcomes appears similar between HIV-positive and HIV negative individuals.[32] However, it may be that HIV-specific risk equations that include HIV-specific risk factors may perform better than existing algorithms because of potential differences in etiology of CVD in the HIV population. For example, D-dimer, a marker of inflammation, has been found to be independently predictive of CVD events. [33] However, the Framingham risk score may partially capture inflammation since markers of inflammation have been found to be associated with a higher score in HIV patients compared with controls [34] and the score has been shown to correlate with the presence of subclinical atherosclerosis measured by carotid artery intima-media thickness in HIV-positive individuals.[35 36] Atherosclerosis may also be high in untreated patients, supporting a role of HIV infection itself as a risk factor.[37]

We used the Framingham risk score because it is based on readily available measures and widely used in clinical practice. Also, our focus was on factors that could be changed by lifestyle interventions, in particular smoking, blood pressure and cholesterol. Alternative scores have used different risk factors, for example, QRISK[38] includes ethnicity and family history of coronary heart disease which are not modifiable, body mass index (BMI), deprivation score, atrial fibrillation, rheumatoid arthritis, and chronic renal disease. Although these risk factors are not entered in the Framingham risk score, they may be important in the evaluation of clinical risk. For example, renal insufficiency has a relatively high prevalence

For peer review only - http://bmjopen.bmj.com/site/about/guidelines.xhtml

in HIV infected black patients and may be an important contributor to risk in this population.[39]

The ability to accurately predict CVD risk is an essential element of this population's care. The Framingham equation for CHD predicted well in The Data Collection on Adverse events of Anti-HIV Drugs Study (D:A:D) in terms of discrimination, but tended to underestimate risk in smokers.[8] The Framingham risk scores may require recalibration to adjust for over or under prediction in the HIV population.[40 41] Factors unique to HIV, such as effects of different antiretroviral drugs, may influence the performance of standard risk prediction tools, as they may change CVD risk both through alterations of traditional risk factors and by contributing to inflammatory and immunologic risk factors. D:A:D found both observed and predicted rates of MI increased with time on ART implying that ART-induced changes in conventional risk factors at least partially explained the increase in risk of MI.[8] The D:A:D predictive model for CVD tailored to HIV patients included exposure to indinavir, boosted lopinavir and abacavir as well as the traditional Framingham risk factors.[9]

The HIV-HEART study found that not only were CVD risk factors high in the HIV population, but also that they are under-treated and therefore risk factor management of HIV patients requires further improvement.[27] The European AIDS Clinical Society (EACS) guidelines on the prevention and management of metabolic disease in HIV [6] state that CVD risk should be assessed in HIV-positive individuals at regular intervals. They recommend lifestyle interventions should focus on counselling to stop smoking, modify diet and take regular exercise. A healthy diet, exercise and maintaining normal body weight tend to reduce dyslipidemia.

For peer review only - http://bmjopen.bmj.com/site/about/guidelines.xhtml

Smoking

Increased Framingham risk scores have been found in HIV-positive compared with negative controls, but this has mostly been due to higher prevalence of smoking rather than to higher cholesterol.[42] Smoking is the most important modifiable risk factor. A pilot study of a smoking cessation programme using counselling and nicotine replacement therapy in the Swiss HIV Cohort Study [43] found that implementing a smoking cessation programme was feasible in HIV-positive individuals. The D:A:D study found that the adjusted incidence rate ratio of CVD decreased from 2.32 within the first year of stopping smoking to 1.49 after greater than 3 years compared with those who never smoked. A recent study from Denmark estimated that HIV-positive individuals lose more years from smoking than from HIV infection.[19]

Future work

In order for accurate CVD risk assessment to be carried out, HIV cohorts need to collect better information on Framingham risk factors and, in particular, on current smoking status of patients. Our study should be considered as a pilot study for assessing heart age. Ideally, this type of analysis would need to be rolled out into a much larger national cohort to properly gauge which factors predict heart age deviation. In addition, CVD events and death would allow us to test whether the Framingham equations are transferable to the UK HIV population. We need to assess the use of heart age as a communication tool for behaviour intervention. More research is required to investigate whether Framingham risk estimates accurately translate to actual CVD events and deaths or whether including HIV specific risk

For peer review only - http://bmjopen.bmj.com/site/about/guidelines.xhtml

factors in the CVD risk equations might result in a more accurate prediction tool for this population.

Implications and conclusion

HIV-positive individuals in this cohort had a considerably increased risk of CVD compared with the ideal reference values. Our research, which showed that heart age exceeds real age at all ages in men and above age 40 years old in women, implies that it is important to estimate CVD risk in HIV-positive individuals. The effect of smoking is to increase heart age on average by 8 to 17 years in males and 8 to 18 years in females depending on the mean real age. This indicates the importance of smoking cessation for prevention of CVD in this population. Furthermore, since the gap between heart age and real age increased as people go older, it is important to intervene on lifestyle factors such as smoking and obesity at young ages. Cardiovascular risk expressed in terms of an individual's heart age, rather than absolute risk of CVD, may have more impact on patients in programmes aiming to intervene on risk factors. Heart age may be a particularly useful tool in communicating risk to younger patients who are at low absolute risk of CVD. Tracking change in heart age, such as that due to smoking cessation, may provide strong motivation towards life style changes.

For peer review only - http://bmjopen.bmj.com/site/about/guidelines.xhtml

Acknowledgements: We thank all patients, Susan Allan, and the team at Southmead Hospital, Bristol, UK. This work was presented in part at the Eleventh International Congress on Drug Therapy in HIV Infection Glasgow, UK 11-15 November 2012. Abstract available: Davies T et al. Journal of the International AIDS Society 2012, 15(Suppl 4):18152

http://www.jiasociety.org/index.php/jias/article/view/18152 | http://dx.doi.org/10.7448/IAS.15.6.18152

Contributors: MG and MM contributed to study design and interpretation of analyses. TD analysed the data, compiled the tables and graphs and wrote the first draft of the paper. MM supervised statistical analyses. All authors contributed to the writing of the paper, edited, revised and approved the final version of the paper and had full access to all the data in the study. MM had the final responsibility for the decision to submit for publication and is the guarantor.

Funding: Margaret May is funded by the Medical Research Council, UK (grant MR/J002380/1). Teri Davies is funded by the UK Medical Research Council as a PhD student in the Bristol Centre for Systems Biomedicine (BCSBmed) doctoral training centre. The views expressed in this manuscript are those of the researchers and not necessarily those of the Medical Research Council. The funders had no role in carrying out the study or in the decision to submit the manuscript for publication.

Competing interests: none.

Ethical approval: The project was approved by a Multi-centre Research Ethics Committee and by local ethics committees.

Copyright: The Corresponding Author has the right to grant on behalf of all authors and does grant on behalf of all authors, an exclusive licence (or non exclusive for government employees) on a worldwide basis to the BMJ Publishing Group Ltd to permit this article (if accepted) to be published in BMJ editions and any other BMJPGL products and sublicences such use and exploit all subsidiary rights, as set out in our licence.

For peer review only - http://bmjopen.bmj.com/site/about/guidelines.xhtml

Data sharing: no additional data available.

For peer review only

For peer review only - http://bmjopen.bmj.com/site/about/guidelines.xhtml

HIV heart age

Table 1: Patient demographic and clinical characteristics at time of Framingham Score assessment

	Number of Patients N = 749	Percent
**Sex**		
Male	503	67.2
**Transmission risk group**		
Heterosexual	386	51.5
MSM	312	41.7
IDU	23	3.1
Blood Product	7	0.9
Unknown	21	2.8
**Ethnicity**		
Black African	228	30.4
White	441	58.9
Other	80	10.7
**Age (years) (median, IQR)**	42.2	(35.5-49.4)
**Age category (years)**		
18-39	310	41.4
40-49	265	35.4
50-59	118	15.8
60+	56	7.5
**Treatment status**		
On ART	612	81.7
**Started ART pre 01/01/2003**	168	22.4
**Viral load (copies per ml)**		
Treated and suppressed (vl $\leq$ 50)	535	71.4
Treated and unsuppressed	77	10.3
Untreated	137	18.3
**Median cd4 count (mm^3), untreated (IQR)**	477	(334-602)
**Median cd4 count (mm^3), treated (IQR)**	559.5	(409-721)
**cd4 count (mm^3)**		
<500	318	42.5
500 - 749	275	36.7
$\geq$ 750	156	20.8
**Median total cholesterol(mg/dL), (IQR)**	189.1	(162-220)
**Median HDL cholesterol(mg/dL), (IQR)**	50.2	(38.6-61.8)
**Median systolic blood pressure(mmHg), (IQR)**	131	(117-143)

For peer review only - http://bmjopen.bmj.com/site/about/guidelines.xhtml

For peer review only

HIV heart age

Table 2: Age, heart age and heart age deviation (heart age- real age) by age group for male and female smokers and non-smokers.

sex	smoking assumption	real age category (years)	n (%)	mean real age (years)	mean heart age (years)	deviation=heart age-real age (years) mean	(95% CI)
males	non-smoker	18-39	184(37%)	33.5	34.9	1.3	(0.60,2.09)
		40-49	182 (36%)	45.1	48.5	3.3	(2.17,4.53)
		50-59	91 (18%)	53.4	59.5	6.1	(3.85,8.37)
		60-	46 (9%)	66.2	73.0	6.8	(2.19,11.36)
		total	503(100%)	44.3	47.7	3.4	(2.65,4.21)
males	smoker	18-39	184(37%)	33.5	43.3	9.8	(8.78,10.74)
		40-49	182 (36%)	45.1	60.1	15.0	(13.53,16.48)
		50-59	91 (18%)	53.4	73.9	20.4	(17.64,23.24)
		60-	46 (9%)	66.2	89.6	23.3	(18.35,28.29)
		total	503(100%)	44.3	59.2	14.8	(13.82,15.84)
females	non-smoker	18-39	126(51%)	33.1	31.7	-1.4	(-2.93,0.05)
		40-49	83 (34%)	44.1	47.1	3.0	(-0.13,6.10)
		50-59	27 (11%)	55.2	62.9	7.7	(1.54,13.93)
		60-	10 (4%)	65.9	82.2	16.3	(0.07,32.52)
		total	246(100%)	40.6	42.4	1.8	(0.15,3.41)
females	smoker	18-39	126(51%)	33.1	39.9	6.8	(4.87,8.64)
		40-49	83 (34%)	44.1	59.2	15.0	(11.19,18.90)
		50-59	27 (11%)	55.2	78.9	23.7	(15.77,31.55)
		60-	10 (4%)	65.9	99.8	33.9	(16.79,51.01)
		total	246(100%)	40.6	53.1	12.5	(10.42,14.61)

For peer review only

For peer review only - http://bmjopen.bmj.com/site/about/guidelines.xhtml

HIV heart age

Table 3: Crude and mutually adjusted heart age deviation (difference from comparator group*) according to patient characteristics for non-smokers and smokers

	All individuals (N=749)			
	Non smoker Deviation (years) (95% CI)		Smoker Deviation (years) (95% CI)	
**Variable**	**Crude**	**Adjusted**	**Crude**	**Adjusted**
**Female vs Male**	-1.65 (-3.25,-0.05)	-0.96 (-2.52,0.59)	-2.32 (-4.38,-0.25)	-0.72(-2.62,1.18)
**Age category**	-	-	-	-
18-39*	-	-	-	-
40-49	3.02 (1.35,4.69)	3.38 (1.66,5.10)	6.48(4.44,8.52)	6.75(4.65,8.86)
50-59	6.27(4.11,8.43)	6.39(4.17,8.61)	12.64(10.00,15.27)	12.64(9.94,15.35)
60+	8.26(5.36,11.16)	8.87(5.90,11.84)	16.67(13.13,20.20)	17.21(13.59,20.83)
**CD4 count category (cells/mm^3)**	-	-	-	-
<500*	-	-	-	-
500-750	1.91(0.23,3.60)	2.39(0.74,4.03)	2.50(0.34,4.66)	3.01(1.00,5.02)
>=750	4.23(2.23,6.23)	4.28(2.28,6.27)	6.12(3.55,8.68)	5.44(3.01,7.87)
**Viral load category**	-	-	-	-
Treated with suppressed vl*	-	-	-	-
Treated with unsuppressed vl	1.07(-1.44,3.59)	2.84(0.40,5.29)	-0.19(-3.42,3.04)	2.90(-0.08,5.89)
Untreated	-1.39(-3.36,0.58)	0.27(-1.74,2.27)	-3.61(-6.15,-1.07)	-0.28(-2.73,2.17)
**ART start date pre 01/01/2003**	0.18(-1.62,1.99)	-2.28(-4.14,-0.42)	1.98(-0.35,4.30)	-2.43(-4.70,-0.17)
**Constant**	-	-1.26(-3.00,0.49)	-	6.70(4.57,8.82)

For peer review only

For peer review only - http://bmjopen.bmj.com/site/about/guidelines.xhtml

HIV heart age

Figure 1: Box plots of distribution of heart age by age group for female non-smokers (upper left), female smokers (upper right), male non-smokers (lower left), male smokers (lower right). The median real age for each age group is shown in red for comparison. In the box plot, the whiskers include 95% of the distribution and the box includes the middle 50% with the median marked as a line. Outliers are shown as filled circles.

For peer review only

HIV heart age

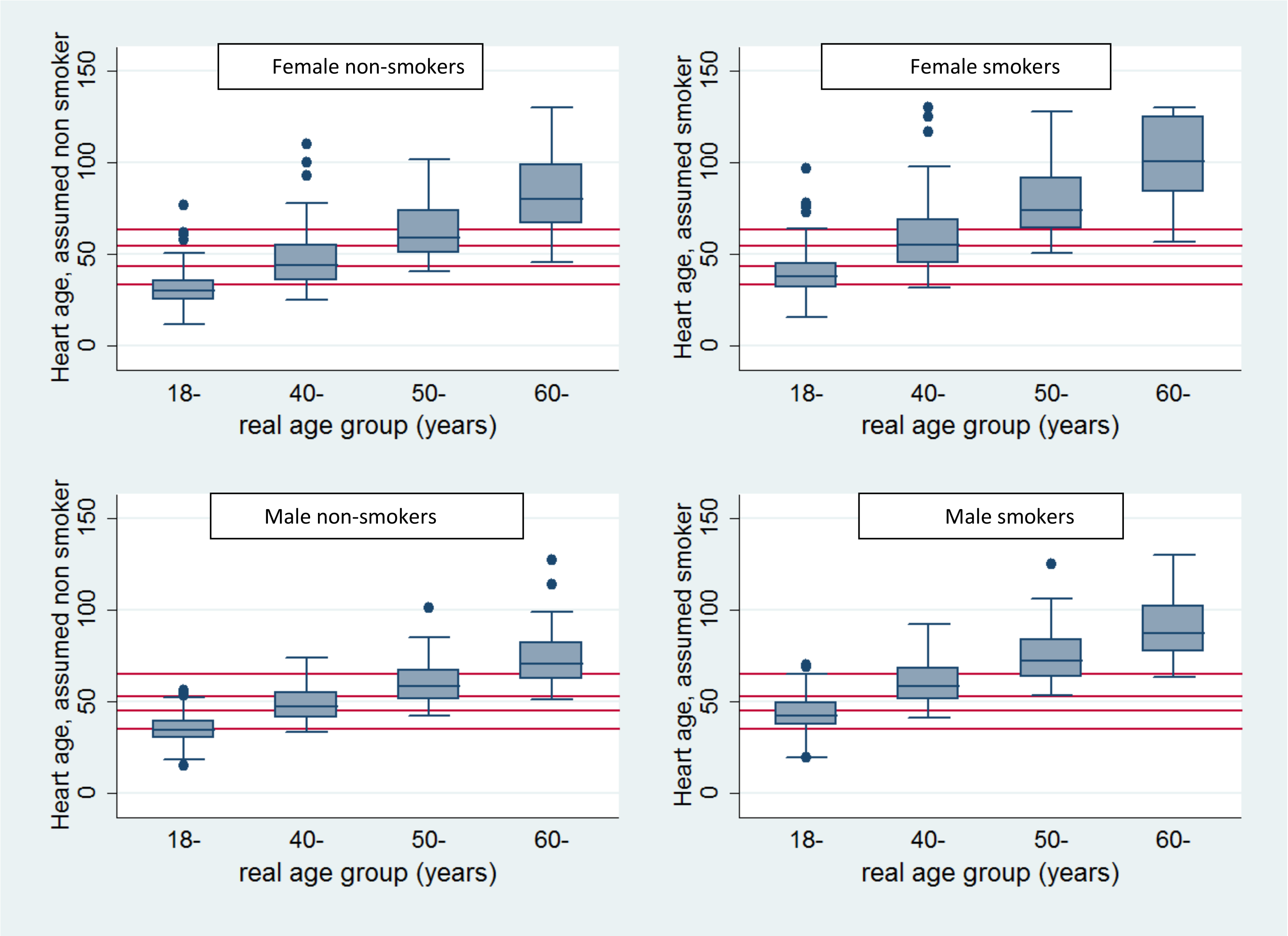

For peer review only - http://bmjopen.bmj.com/site/about/guidelines.xhtml

Figure 2: Heart age deviation in years (95% CI shown shaded in gray) for male and female smokers and non-smokers for real ages 20 to 70 years old

For peer review only

For peer review only - http://bmjopen.bmj.com/site/about/guidelines.xhtml

HIV heart age

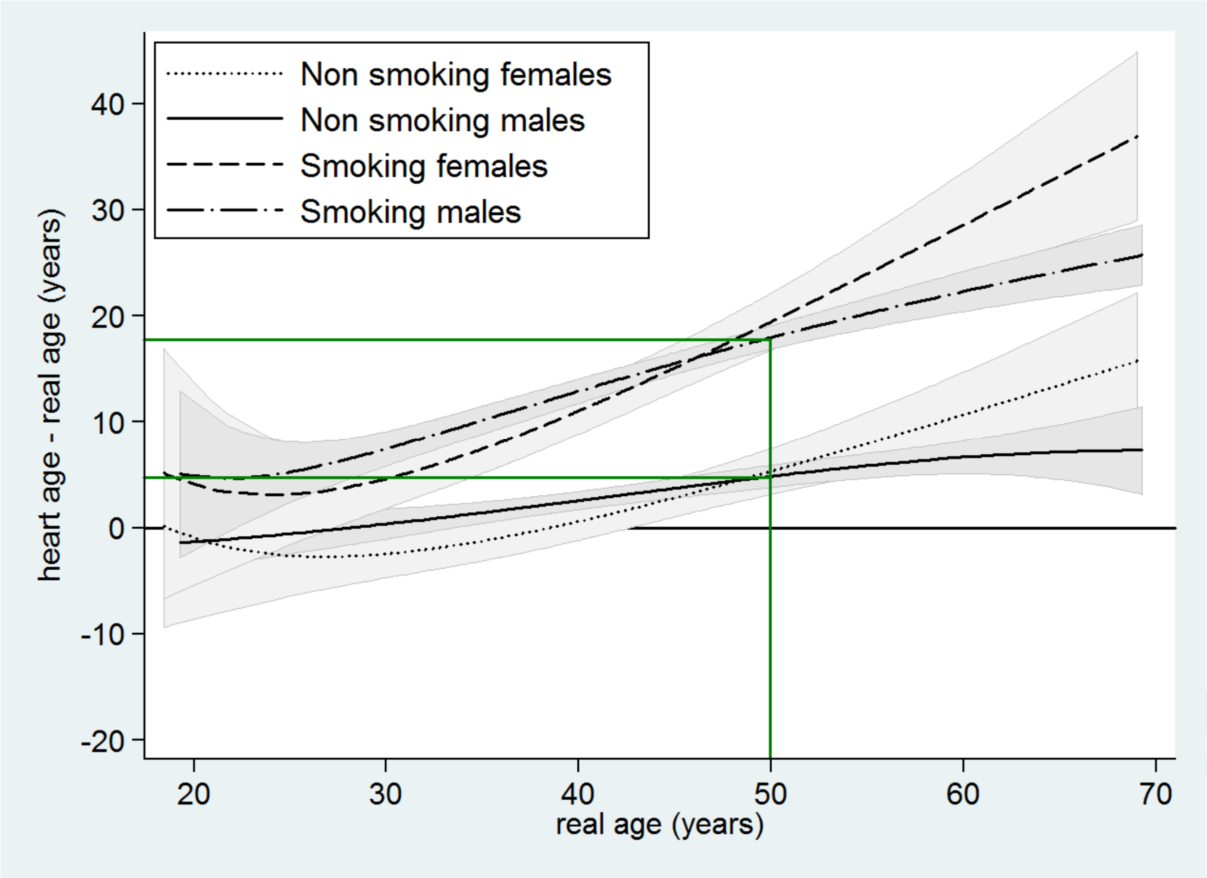

For peer review only - http://bmjopen.bmj.com/site/about/guidelines.xhtml

REFERENCES

1. Canadian Cardiovascular Society. CCS Guideline Programs: Updated CCS Dyslipidemia Guidelines Knowledge Translation Tools., 2013. http://www.ccsguidelineprograms.ca/index.php accessed 02/08/2013.

2. May MT, Ingle SM. Life expectancy of HIV-positive adults: a review. *Sex Health* 2011;8(4):526-33.

3. Nakagawa F, Lodwick RK, Smith CJ, Smith R, Cambiano V, Lundgren J, et al. Projected life expectancy of people with HIV according to timing of diagnosis. *AIDS* 2012;26:335-43.

4. Onen NF, Overton ET, Seyfried W, Stumm ER, Snell M, Mondy K, et al. Aging and HIV infection: a comparison between older HIV-infected persons and the general population. *HIV clinical trials* 2010;11(2):100-9.

5. Law M, Friis-Moller N, Weber R, Reiss P, Thiebaut R, Kirk O, et al. Modelling the 3-year risk of myocardial infarction among participants in the Data Collection on Adverse Events of Anti-HIV Drugs (DAD) study. *HIV.Med* 2003;4(1):1-10.

6. Lundgren JD, Battegay M, Behrens G, De Wit S, Guaraldi G, Katlama C, et al. European AIDS Clinical Society (EACS) guidelines on the prevention and management of metabolic diseases in HIV. *HIV Med* 2008;9(2):72-81.

7. May M, Sterne JA, Shipley M, Brunner E, d'Agostino R, Whincup P, et al. A coronary heart disease risk model for predicting the effect of potent antiretroviral therapy in HIV-1 infected men. *International journal of epidemiology* 2007;36(6):1309-18.

8. Law MG, Friis-Moller N, El-Sadr WM, Weber R, Reiss P, D'Arminio MA, et al. The use of the Framingham equation to predict myocardial infarctions in HIV-infected patients: comparison with observed events in the D:A:D Study. *HIV.Med.* 2006;7(4):218-30.

9. Friis-Moller N, Thiebaut R, Reiss P, Weber R, Monforte AD, De WS, et al. Predicting the risk of cardiovascular disease in HIV-infected patients: the Data collection on Adverse Effects of Anti-HIV Drugs Study. *Eur J Cardiovasc Prev Rehabil.* 2010.

10. D'Agostino RB, Sr., Vasan RS, Pencina MJ, Wolf PA, Cobain M, Massaro JM, et al. General cardiovascular risk profile for use in primary care: the Framingham Heart Study. *Circulation* 2008;117(6):743-53.

11. Burke EG, Nelson J, Kwong J, Cook PF. Cardiovascular risk assessment for persons living with HIV. *The Journal of the Association of Nurses in AIDS Care : JANAC* 2012;23(2):134-45.

12. Framingham Heart Study. General Cardiovascular Disease (10-year risk), 2011 http://www.framinghamheartstudy.org/risk/gencardio.html Accessed 02/08/2013

13. Royston P, Ambler G, Sauerbrei W. The use of fractional polynomials to model continuous risk variables in epidemiology. *Int.J.Epidemiol.* 1999;28(5):964-74.

14. Stata Statistical Software: Release 12 [program]. College Station, TX: StataCorp LP., 2011.

15. Health Survey for England - 2006, CVD and risk factors for adults, obesity and risk factors for children 2008. Available from: www.ic.nhs.uk/pubs/HSE06CVDandriskfactors. accessed 15/01/2013

16. Triant VA, Lee H, Hadigan C, Grinspoon SK. Increased acute myocardial infarction rates and cardiovascular risk factors among patients with human immunodeficiency virus disease. *The Journal of clinical endocrinology and metabolism* 2007;92(7):2506-12.

17. Petoumenos K, Worm SW. HIV infection, aging and cardiovascular disease: epidemiology and prevention. *Sex Health* 2011;8(4):465-73.

18. Smith CJ, Levy I, Sabin CA, Kaya E, Johnson MA, Lipman MC. Cardiovascular disease risk factors and antiretroviral therapy in an HIV-positive UK population. *HIV Med* 2004;5(2):88-92.

19. Helleberg M, Afzal S, Kronborg G, Larsen CS, Pedersen G, Pedersen C, et al. Mortality Attributable to Smoking Among HIV-1-Infected Individuals: A Nationwide, Population-Based Cohort Study. *Clin Infect Dis* 2013;56(5):727-34.

20. Neergaard L. Aging AIDS epidemic raises new questions. *NBC News*, 2012. Available from: http://www.nbcnews.com/id/48346557/ns/health-aids/#.USd556XvioZ. accessed 15/01/2013

For peer review only - http://bmjopen.bmj.com/site/about/guidelines.xhtml

21. El-Sadr WM, Lundgren JD, Neaton JD, Gordin F, Abrams D, Arduino RC, et al. CD4+ count-guided interruption of antiretroviral treatment. *N.Engl.J.Med.* 2006;355(22):2283-96.
22. De Socio GV, Martinelli C, Ricci E, Orofino G, Valsecchi L, Vitiello P, et al. Relations between cardiovascular risk estimates and subclinical atherosclerosis in naive HIV patients: results from the HERMES study. *Int J STD AIDS* 2010;21(4):267-72.
23. Guaraldi G, Zona S, Alexopoulos N, Orlando G, Carli F, Ligabue G, et al. Coronary aging in HIV-infected patients. *Clin Infect Dis* 2009;49(11):1756-62.
24. Frostegard J, Ulfgren AK, Nyberg P, Hedin U, Swedenborg J, Andersson U, et al. Cytokine expression in advanced human atherosclerotic plaques: dominance of pro-inflammatory (Th1) and macrophage-stimulating cytokines. *Atherosclerosis* 1999;145(1):33-43.
25. Maggi P, Perilli F, Lillo A, Carito V, Epifani G, Bellacosa C, et al. An ultrasound-based comparative study on carotid plaques in HIV-positive patients vs. atherosclerotic and arteritis patients: atherosclerotic or inflammatory lesions? *Coronary artery disease* 2007;18(1):23-9.
26. Zhou X, Nicoletti A, Elhage R, Hansson GK. Transfer of CD4(+) T cells aggravates atherosclerosis in immunodeficient apolipoprotein E knockout mice. *Circulation* 2000;102(24):2919-22.
27. Reinsch N, Neuhaus K, Esser S, Potthoff A, Hower M, Mostardt S, et al. Are HIV patients undertreated? Cardiovascular risk factors in HIV: results of the HIV-HEART study. *European journal of preventive cardiology* 2012;19(2):267-74.
28. D'Agostino RB, Sr. Cardiovascular risk estimation in 2012: lessons learned and applicability to the HIV population. *The Journal of infectious diseases* 2012;205 Suppl 3:S362-7.
29. Knobel H, Jerico C, Montero M, Sorli ML, Velat M, Guelar A, et al. Global cardiovascular risk in patients with HIV infection: concordance and differences in estimates according to three risk equations (Framingham, SCORE, and PROCAM). *AIDS patient care and STDs* 2007;21(7):452-7.
30. Guimaraes MMM, Greco DB, Garces AHI, de Oliveira AR, Foscolo RB, Machado LJD. Coronary heart disease risk assessment in HIV-infected patients: a comparison of Framingham, PROCAM and SCORE risk assessment functions. *Int J Clin Pract* 2010;64(6):739-45.
31. Barros ZM, de Alencar Ximenes RA, Miranda-Filho DB, de Albuquerque Mde F, Melo HR, Carvalho EH, et al. Comparison between the Framingham and prospective cardiovascular of Munster scores for risk assessment of coronary heart disease in human immunodeficiency virus-positive patients in Pernambuco, Brazil. *Metabolic syndrome and related disorders* 2010;8(6):489-97.
32. Schambelan M, Wilson PW, Yarasheski KE, Cade WT, Davila-Roman VG, D'Agostino RB, Sr., et al. Development of appropriate coronary heart disease risk prediction models in HIV-infected patients. *Circulation* 2008;118(2):e48-53.
33. Ford ES, Greenwald JH, Richterman AG, Rupert A, Dutcher L, Badralmaa Y, et al. Traditional risk factors and D-dimer predict incident cardiovascular disease events in chronic HIV infection. *AIDS* 2010;24(10):1509-17.
34. Guzman-Fulgencio M, Medrano J, Rallon N, Echeverria-Urabayen A, Benito JM, Restrepo C, et al. Soluble markers of inflammation are associated with Framingham scores in HIV-infected patients on suppressive antiretroviral therapy. *J Infection* 2011;63(5):382-90.
35. Parra S, Coll B, Aragones G, Marsillach J, Beltran R, Rull A, et al. Nonconcordance between subclinical atherosclerosis and the calculated Framingham risk score in HIV-infected patients: relationships with serum markers of oxidation and inflammation. *HIV Med* 2010;11(4):225-31.
36. Falcone EL, Mangili A, Skinner S, Alam A, Polak JF, Wanke CA. Framingham risk score and early markers of atherosclerosis in a cohort of adults infected with HIV. *Antivir Ther* 2011;16(1):1-8.
37. Maggi P, Quirino T, Ricci E, De Socio GV, Gadaleta A, Ingrassia F, et al. Cardiovascular risk assessment in antiretroviral-naive HIV patients. *AIDS patient care and STDs* 2009;23(10):809-13.

For peer review only

38. Hippisley-Cox J, Coupland C, Vinogradova Y, Robson J, Minhas R, Sheikh A, et al. Predicting cardiovascular risk in England and Wales: prospective derivation and validation of QRISK2. *BMJ.* 2008;336(7659):1475-+.
39. Campbell LJ, Desai M, Hegazi A, Ibrahim F, Melikian N, Hay P, et al. Renal Impairment Is Associated With Coronary Heart Disease in HIV-Positive Men. *HIV clinical trials* 2012;13(6):343-49.
40. D'Agostino RB, Sr., Grundy S, Sullivan LM, Wilson P, Group CHDRP. Validation of the Framingham coronary heart disease prediction scores: results of a multiple ethnic groups investigation. *Journal of the American Medical Association* 2001;286(2):180-87.
41. Brindle PM, McConnachie A, Upton MN, Hart CL, Davey Smith G, Watt GC. The accuracy of the Framingham risk-score in different socioeconomic groups: a prospective study. *Br J Gen Pract* 2005;55(520):838-45.
42. De Socio GV, Parruti G, Quirino T, Ricci E, Schillaci G, Adriani B, et al. Identifying HIV patients with an unfavorable cardiovascular risk profile in the clinical practice: results from the SIMONE study. *The Journal of infection* 2008;57(1):33-40.
43. Elzi L, Spoerl D, Voggensperger J, Nicca D, Simcock M, Bucher HC, et al. A smoking cessation programme in HIV-infected individuals: a pilot study. *Antivir Ther* 2006;11(6):787-95.

For peer review only

For peer review only - http://bmjopen.bmj.com/site/about/guidelines.xhtml

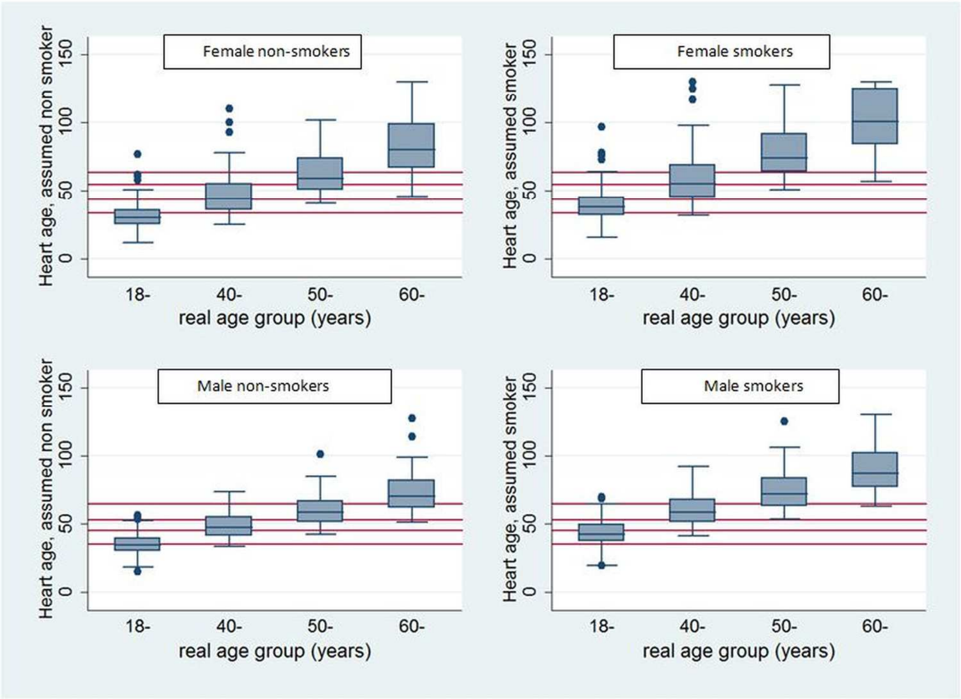

228x165mm (300 x 300 DPI)

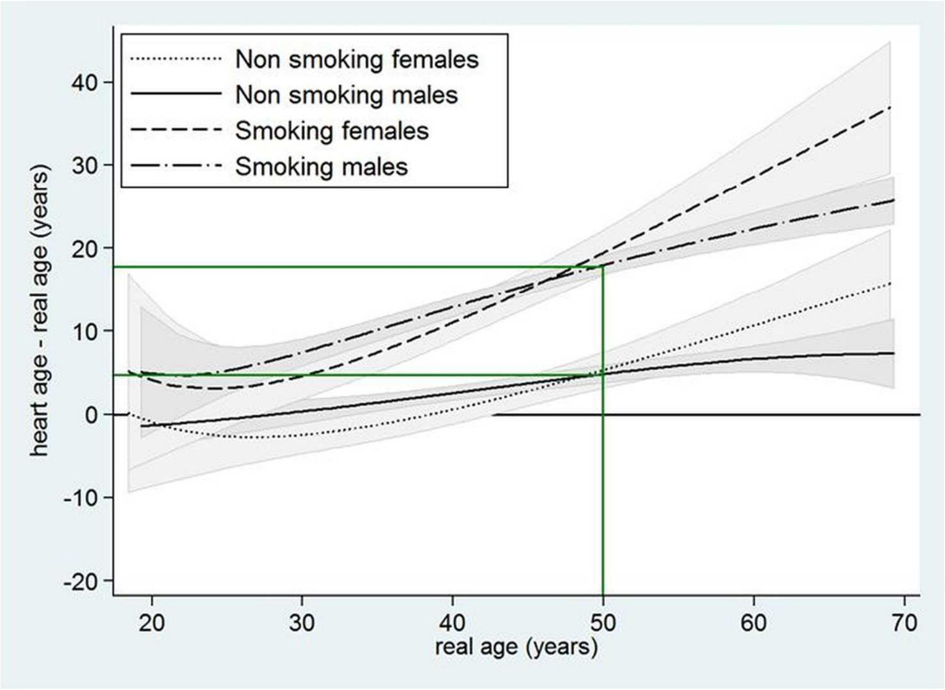

198x144mm (300 x 300 DPI)
